# The management of iron deficiency in inflammatory bowel disease – an online tool developed by the RAND/UCLA appropriateness method

**DOI:** 10.1111/apt.12493

**Published:** 2013-09-17

**Authors:** W Reinisch, Y Chowers, S Danese, A Dignass, F Gomollón, O Haagen Nielsen, P L Lakatos, C W Lees, S Lindgren, M Lukas, G J Mantzaris, P Michetti, B Moum, L Peyrin-Biroulet, M Toruner, J Woude, G Weiss, H Stoevelaar

**Affiliations:** * Department Internal Medicine III, Medical University of ViennaVienna, Austria; † Department of Gastroenterology, Rambam Health Care CampusHaifa, Israel; ‡ Department of Gastroenterology, Humanitas Clinical and Research CenterMilan, Italy; § Department of Gastroenterology, Oncology, Infectious Diseases and Metabolism, Agaplesion Markus HospitalFrankfurt, Germany; ¶ Department of Gastroenterology, Hospital Clínico Universitario Lozano Blesa, CIBEREHDZaragoza, Spain; ** Department of Gastroenterology, Herlev Hospital, University of CopenhagenCopenhagen, Denmark; †† 1^st^ Department of Medicine, Semmelweis UniversityBudapest, Hungary; ‡‡ Department of Gastroenterology, Western General HospitalEdinburgh, UK; §§ Department of Gastroenterology, University Hospital Skane, University of LundMalmö, Sweden; ¶¶ IBD Clinical and Research Centre, ISCARE Lighthouse and 1^st^ Medical Faculty, Charles UniversityPrague, Czech Republic; *** 1^st^ Department of Gastroenterology, Evangelismos HospitalAthens, Greece; ††† Department of Gastroenterology, Lausanne University Medical CenterLausanne, Switzerland; ‡‡‡ Department of Gastroenterology, Oslo University Hospital, University of OsloOslo, Norway; §§§ Inserm, U954 and Department of Hepato-Gastroenterology, University Hospital of Nancy, Université Henri Poincaré 1Vandoeuvre-lès-Nancy, France; ¶¶¶ Department of Gastroenterology, Ankara University School of MedicineAnkara, Turkey; **** Department of Gastroenterology and Hepatology, Erasmus University Medical CenterRotterdam, The Netherlands; †††† Department of Internal Medicine, Clinical Immunology and Infectious Diseases, Medical University of InnsbruckInnsbruck, Austria; ‡‡‡‡ Center for Decision Analysis and Support, Ismar HealthcareLier, Belgium

## Abstract

**Background**Iron deficiency is a common and undertreated problem in inflammatory bowel disease (IBD).

**Aim**To develop an online tool to support treatment choice at the patient-specific level.

**Methods**Using the RAND/UCLA Appropriateness Method (RUAM), a European expert panel assessed the appropriateness of treatment regimens for a variety of clinical scenarios in patients with non-anaemic iron deficiency (NAID) and iron deficiency anaemia (IDA). Treatment options included adjustment of IBD medication only, oral iron supplementation, high-/low-dose intravenous (IV) regimens, IV iron plus erythropoietin-stimulating agent (ESA), and blood transfusion. The panel process consisted of two individual rating rounds (1148 treatment indications; 9-point scale) and three plenary discussion meetings.

**Results**The panel reached agreement on 71% of treatment indications. ‘No treatment’ was never considered appropriate, and repeat treatment after previous failure was generally discouraged. For 98% of scenarios, at least one treatment was appropriate. Adjustment of IBD medication was deemed appropriate in all patients with active disease. Use of oral iron was mainly considered an option in NAID and mildly anaemic patients without disease activity. IV regimens were often judged appropriate, with high-dose IV iron being the preferred option in 77% of IDA scenarios. Blood transfusion and IV+ESA were indicated in exceptional cases only.

**Conclusions**The RUAM revealed high agreement amongst experts on the management of iron deficiency in patients with IBD. High-dose IV iron was more often considered appropriate than other options. To facilitate dissemination of the recommendations, panel outcomes were embedded in an online tool, accessible via http://ferroscope.com/.

## Introduction

Iron deficiency is a common condition in patients with inflammatory bowel disease (IBD) and may be caused by intestinal blood loss, reduced duodeno-jejunal iron absorption and/or dietary restrictions.^1^ Prevalence data range from 36% to 90%, depending on the study population and definitions used.^2^ Iron deficiency is one of the major causes of anaemia in IBD (iron deficiency anaemia; IDA), having a significant impact on the patient's quality of life.^3^–^4^ This impact is not only seen in patients with clinical symptoms of anaemia, but could also be ‘hidden’ in slowly deteriorating physical and cognitive function, often not readily recognised by patients and their physicians.^5^–^6^ Several studies have shown that correcting iron deficiency in IBD positively affects the patient's quality of life.^3^,^4^ Evidence from other disease areas supports this beneficial impact to be also present in patients with non-anaemic iron deficiency (NAID).^8^–^9^ There are various treatment options available for correcting iron deficiency and anaemia and many new formulations have been introduced over the last few years. These include oral iron supplementation and intravenous (IV) regimens, the latter in low or high doses. For selected patient groups, other regimens may be considered as well, such as adjustment of IBD medication, addition of an erythropoietin-stimulating agent to IV iron (IV+ESA), and blood transfusion. Treatment choice requires carefully balancing the benefits and negative consequences of the available treatments in relation to specific patient conditions such as the presence of symptoms, IBD disease activity and haemoglobin levels.^1^ It is also important to take into consideration that anaemia in IBD may be caused by other factors besides iron deficiency. Inflammation-induced changes to the iron metabolism and erythropoiesis form the second important cause of anaemia in IBD (anaemia of chronic disease; ACD). Other less common causes are cobalamin and folate deficiency and use of particular drugs.^1^ IDA and ACD are frequently overlapping conditions. Distinguishing between the causes of anaemia in IBD is important from a therapeutic perspective: iron supplementation may be unnecessary or even counterproductive in pure ACD, while it is mostly necessary in IDA.^10^–^11^ Paradoxically, despite their high prevalence in IBD, anaemia and iron deficiency are believed to be underdiagnosed, neglected as a clinical problem and often undertreated.^2^ This could partly be ascribed to the lack of or slow development of clinical symptoms, but has also been attributed to a low awareness amongst gastroenterologists because they are ‘commonly exposed to severe blood loss and low haemoglobin levels’.^1^

To support adequate management of iron deficiency and anaemia in IBD, Gasché *et al*. developed a guideline specifically dedicated to this topic.^1^ In addition, recommendations and treatment algorithms have been included in guidelines on IBD^12^,^13^ and IDA^15^, and also in some review articles.^16^–^17^ All guidelines and recommendations express the need of increased attention, timely diagnosis and appropriate treatment choice, but differ in their level of specificity, scope and views on the mode of administration. To streamline the available evidence in the light of the heterogeneity of IBD patients, a European group of gastroenterologists initiated a project to develop recommendations on the treatment of NAID/IDA in IBD at the patient-specific level, and to make these recommendations available to practising physicians via an online decision support tool.

## Methods

### Study design

**Figure 1:**
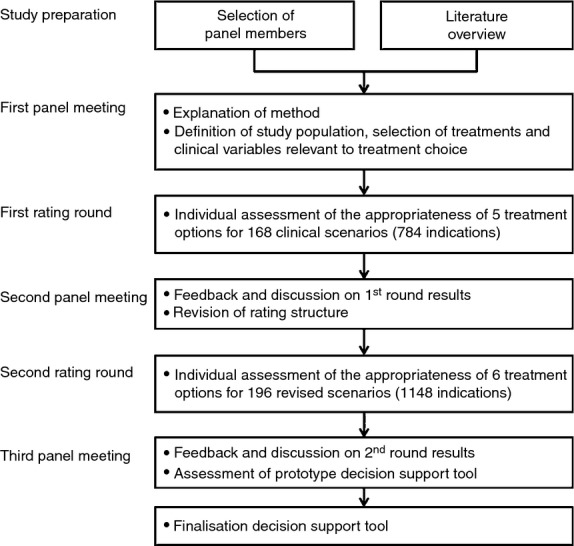
Study design.

To develop treatment recommendations at the patient-specific level, we used the RAND/UCLA Appropriateness Method (RUAM).^18^,^19^ This modified Delphi method has been widely applied to determine the appropriateness of medical and surgical procedures in various fields of medicine, including several aspects of the management of IBD.^21^–^24^ The RUAM provides a structured approach to combine best evidence from clinical studies with the collective judgments of a panel of experts. Several studies have shown that the RUAM produces reliable, internally consistent and clinically valid results.^20^–^29^ The study design is depicted in Figure 1.

### Panel composition

The panel consisted of 16 gastroenterologists from 16 European countries, and a (nonvoting) expert in iron deficiency and anaemia (GW). Selection of the panel members was done by the panel chair (WR) on the basis of their clinical and scientific expertise in the field of IBD, and geographical spread to allow a European view on the topic. The panel was supported by a methodologist experienced in the RUAM (HS).

### Literature study

A comprehensive literature study was conducted by a methodologist experienced in the RUAM to serve two purposes. First, the results were used to shape the research question and to determine the study design. Secondly, a comprehensive overview of clinical studies was provided as unbiased as possible to the panellists, ensuring that they had access to the same body of evidence while doing the appropriateness assessments (see Data S1). In addition, the first author performed independently an in-depth review on the topic of iron deficiency in IBD.^30^

### Panel process and rating structure

During the first panel meeting (October 2011), the results of the literature research were discussed, extended and refined. The final results were included in the electronic rating tool as look-up materials (see Data S1). Furthermore, the starting points of the study were determined.

The study population was defined as patients with IBD (newly diagnosed, or at any follow-up), having biochemical evidence of iron deficiency, with or without physical signs and symptoms. Patients with conditions likely to be the primary cause of anaemia, other than iron deficiency and ACD, were excluded from the considerations; additional exclusion criteria were age <18 years, pregnancy and presence of absolute contra-indications for any of the treatments included. Because of the presumed difference in therapeutic approach, the patient population was split into two groups according to the presence of anaemia: NAID and IDA. Selected treatment options for NAID included no active treatment, oral iron, and IV regimens (low/high dose; cut-off point 500 mg iron per single infusion). For IDA, the combination of an IV regimen plus ESA was added to the therapeutic arsenal. For both NAID and IDA, the panel selected a set of clinical variables considered relevant to treatment choice, such as previous treatment of iron deficiency, and symptoms and conditions related to iron deficiency and anaemia. By permutation, a set of 56 different scenarios was constructed for NAID, and 112 scenarios for IDA. Panellists used an electronic rating program to individually assess the appropriateness of treatments for all scenarios on a 9-point scale (reference values: 1 = inappropriate, 5 = uncertain, 9 = appropriate). They were instructed to consider only the clinical perspective and to disregard financial costs and other potential constraints to the availability of treatments. During a plenary meeting (March 2012), the results of the first rating round were discussed. The discussion revealed some differences in the interpretation of the definitions and clinical scenarios, and also in the way the appropriateness of treatments had been rated. In addition, the panel concluded that particular treatment options were missing. Subsequently, a second individual rating round took place, using an adapted structure and refined definitions and instructions. This round included the assessment of 1148 indications spread over 196 clinical scenarios. An overview of clinical variables and treatment options is provided in Table 1. Based on the second round results, a prototype of an electronic decision support tool was developed. During a third panel meeting (October 2012), final recommendations and adaptations to the tool were established.

**Table 1:** Overview of variables and treatment options used for the construction of clinical scenarios in the second rating round (for details: see appendix 1)

	NAID	IDA
Variables		
Previous treatment of iron deficiency	X	X
Haemoglobin level		X
Physical symptoms of iron depletion		X
Conditions associated with additional iron need	X	X
IBD activity status	X	X
Treatment options		
None	X	X
Adjusting IBD medication only	X	
Oral iron	X	X
Low-dose IV iron	X	X
High-dose IV iron	X	X
IV iron + ESA		X
Blood transfusion		X

IBD, inflammatory bowel disease; IV, intravenous; ESA, erythropoietin-stimulating agent; NAID, non-anaemic iron deficiency; IDA, iron deficiency anaemia.

### Statistical analysis

The translation of individual ratings to panel statements was based on the mathematical rules that are typically applied in RUAM studies.^19^ The panel was said to have agreement if at least 12 out of the 16 ratings were in the same section of the 9-point scale (1–3, 4–6 or 7–9). Disagreement was defined as the situation in which at least 5 panellists scored in each of the sections 1–3 and 7–9. An indication was deemed appropriate if the median panel score was between 7 and 9, and inappropriate if the median was between 1 and 3, both without disagreement. All other outcomes were labelled ‘uncertain’. Frequency tables and cross-tabulations were used to describe and analyse the appropriateness of treatments in relation to patient conditions.

## Results

### Agreement

Overall, agreement increased from 43% in the first round to 71% in the second round. Disagreement (12% after the second round) was largely explained by different views on the use of low-dose IV iron in patients with IDA.

### Appropriateness

Appropriateness figures after the second round are summarised in Table 2.

**Table 2:** Appropriateness figures (%) after the second round (sum of row totals is 100%)

Treatment	Inappropriate	Uncertain	Appropriate
Non-anaemic iron deficiency			
None	96	4	0
Adjustment IBD medication	50	0	50
Oral iron	71	18	11
Low-dose IV iron	43	29	29
High-dose IV iron	0	32	68
Iron deficiency anaemia			
None	100	0	0
Oral iron	87	12	1
Low-dose IV iron	43	50	7
High-dose IV iron	1	14	86
IV iron + ESA	82	5	14
Blood transfusion	77	21	1

IBD, inflammatory bowel disease; IV, intravenous; ESA, erythropoietin-stimulating agent.

Providing no treatment was almost always deemed inappropriate. For both NAID and IDA, IV iron was the treatment option most frequently considered appropriate, with high-dose IV iron being the preferred option in 77% of IDA scenarios. For almost all scenarios (98%), there was at least one appropriate option available, while exactly one appropriate option existed for 83% of scenarios.

### Factors determining the appropriateness of treatment

**Figure 2:**
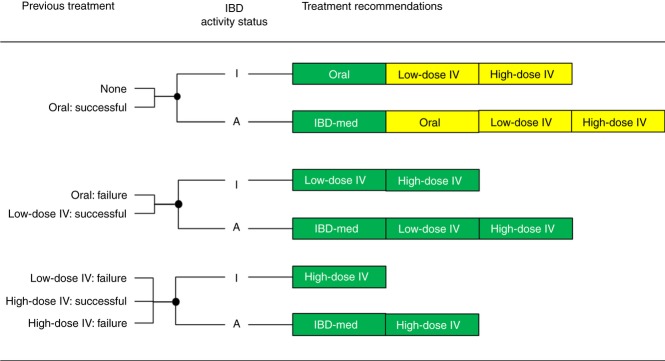
Summarised recommendations for NAID by previous treatment and IBD activity status. Appropriate treatments are displayed in green, optional treatments (uncertain or appropriate in specific situations) in yellow. IBD activity status: I = inactive, A = active; IBD-med: adjustment of IBD medication only; IV: intravenous.

Cross-tabulations showed that previous treatment was the most discriminative factor for the appropriateness outcomes, followed by IBD activity status for NAID, and haemoglobin level for IDA. Recommendations by principal variables are summarised in Figure 2 for NAID. A distinction was made between appropriate and optional, the latter consisting of indications for which the outcome was uncertain for most cases, or appropriate in only very specific subgroups. Oral iron was considered appropriate in inactive disease if there was no previous treatment or if oral iron had been successful in the past. IBD activity status (inactive/active) showed a clear-cut outcome for the adjustment of IBD medication only. In patients with active disease, this treatment option was always appropriate, while being inappropriate in those with inactive disease. Low-dose IV iron was deemed appropriate if previous use had been successful, and also after failure of oral treatment. High-dose IV iron was considered appropriate for the majority of NAID cases, even if previous use had not been successful.

**Figure 3:**
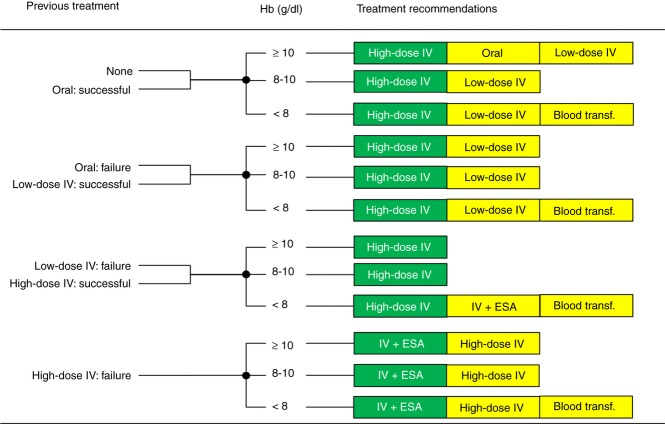
Summarised recommendations for IDA by previous treatment and haemoglobin level. Appropriate treatments are displayed in green, optional treatments (uncertain or appropriate in specific situations) in yellow. Hb: haemoglobin level; IV: intravenous.

Summarised recommendations for IDA are provided in Figure 3. High-dose IV iron was almost always considered an appropriate treatment, except when it had previously failed. In those cases, IV iron + ESA was the preferred option. Low-dose IV iron was judged appropriate if it had successfully been used in the past. Blood transfusion was usually deemed inappropriate, but the outcomes indicate that it could be an option in selected patients with a haemoglobin level below 8 g/dl, for example, in those having physical symptoms of anaemia.

### Online decision support tool

**Figure 4:**
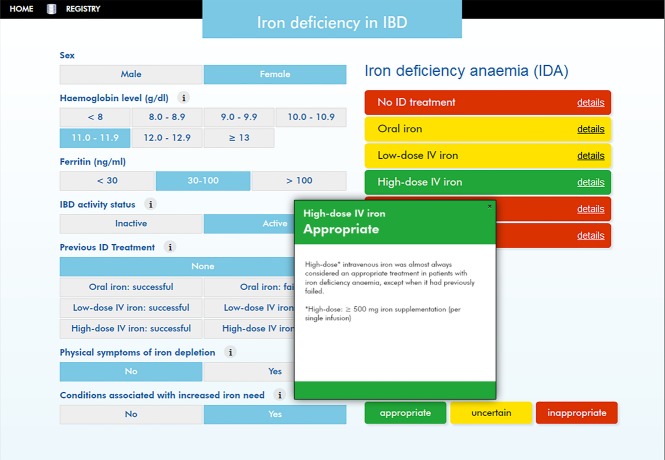
User interface of the online decision support tool. An example of a patient profile is highlighted in blue at the left side. The appropriateness of treatment options under consideration is visualised by colour codes at the right side. Panel considerations (for high-dose IV iron in this example) are available by clicking on ‘details’.

Figures 2 and 3 cover the global panel outcomes in relation to the most important clinical variables. However, for a number of indications, the appropriateness patterns were more complex, and full representation is almost impossible in a paper form. Therefore, data were embedded in an online decision support tool that allows the user to select a patient profile and to view the appropriateness of treatments for that profile, including the panel considerations behind the outcomes (Figure 4). Similar tools have been developed for other topics related to the management of IBD.^21^,^22^ The program can be accessed via http://ferroscope.com/.

## Discussion

It is increasingly recognised that iron deficiency and IDA are underdiagnosed and undertreated conditions in patients with IBD.^2^,^31^ This has been attributed not only to a lack of awareness about the impact of these conditions on quality of life^1^, but also to common misconceptions about their prevalence, and about the efficacy and safety of the available iron supplementation regimens.^2^ In addition, the heterogeneity and complexity of iron deficiency and anaemia in IBD patients necessitate a tailored approach in relation to relevant patient characteristics.^1^ The RUAM proved to be useful in exploring the appropriateness of treatments for a variety of clinical scenarios and in producing recommendations at the patient-specific level.

The expert panel reached high agreement on the appropriateness of 1148 treatment indications spread over 196 clinical scenarios. The panel results were both comprehensive and specific. In 98% of scenarios, there was at least one treatment option considered appropriate, and in 83%, this concerned exclusively one of the treatments.

The RUAM focused on classes of formulations as oral, low-dose IV and high-dose IV iron instead of specific compounds. Oral iron treatments may have some slight pharmacokinetics differences depending on the iron salt used, the iron content or the combinations with additives such as ascorbic acid. However, there are no reliable evidence-based data comparing different oral preparations so far. The same applies within the classes of low-dose and high-dose iron formulations. In addition, available IV iron formulations have a similarly excellent safety profile with an extremely low risk for serious adverse events.^30^

For both NAID and IDA, ‘no treatment’ was generally considered inappropriate. Current guidelines and recommendations also stress that treatment should be considered for all patients with IDA^1^,^12^, but are not or less explicit for treatment initiation in NAID. The guidelines provided by Gasché *et al*. mention that the decision on iron supplementation in NAID is more complicated and that different approaches should be considered.^1^ According to the algorithm developed by Munoz *et al*., iron supplementation is also recommended in patients with NAID.^16^ Adjustment of IBD treatment only (thus without iron supplementation) was considered in our study only as a treatment option for patients with NAID. The outcomes here were clear-cut, recommending this as an appropriate option in all patients with active disease. Scenarios for which oral iron was considered appropriate were primarily related to NAID. In IDA, it is an appropriate option in patients after previously successful oral treatment, but not if the haemoglobin level is less than 10 g/dl. For most indications, IV iron was recommended. This is in line with the guideline by Gasché *et al*. who stated that IV iron is the preferred route of supplementation, although many patients respond to oral iron.^1^ The rationale behind this statement is the often higher efficacy and better tolerance of IV regimens in IBD.^1^ The IBD guideline of the British Society of Gastroenterology takes a different perspective, mentioning that oral supplementation can be used as an initial therapy, and that IV regimens are preferred in patients with poor tolerance to oral iron.^14^ This does not take into account that dietary iron absorption is negatively affected by inflammation via the action of cytokines and the iron regulatory peptide hepcidin,^10^,^11^ suggesting that oral iron is less absorbed in IBD patients with inflammatory activity. The algorithm of Munoz *et al*. follows this approach, but advices immediate IV iron in patients with a haemoglobin level <10 g/dL.^16^

In our study, high-dose IV iron (>500 mg iron per single infusion) was more often judged appropriate than lower doses (68% vs. 29% in NAID; 86% vs. 7% in IDA). In the FERGICOR study, which compared two different treatment strategies including a low-dose IV iron sucrose (IS) and high-dose IV ferric carboxymaltose (FC) arm in patients with IBD, a superior cost-effectiveness of high-dose IV was suggested.^34^ More infusions were required in the IS group and total treatment costs for FC over the whole study period were lower.^34^ A retrospective study from Denmark also suggested that FC was more cost-effective as compared with IS due to fewer out-patient visits. The corresponding Budget Impact Analysis from a hospital perspective showed that FC was more expensive.^35^ The collective ratings from the panel obviously share the opinion that regimens that allow administration of the full dose at one time or in few sessions only are much more convenient to the patient, by preventing repeat visits and avoiding disturbance of activities of daily life. However, as in almost all RUAM studies, costs of treatment were not taken into consideration and the panel did not weigh the usually higher costs of high-dose IV iron regimens vs. their benefits for the patient and reduced healthcare utilisation.

The panel reserved IV iron + ESA predominantly for patients insufficiently responding to high-dose IV iron which is in accordance with the Gasché guideline.^1^ However, such patients may need a careful re-evaluation to rule out other causes for anaemia or nonresponse to IV iron.^36^–^37^ Adequate diagnosis and treatment of iron deficiency should avoid blood transfusion^1^, and only few scenarios existed for which the panel deemed this to be an appropriate option. These concerned specific cases of anaemia with a haemoglobin level <8 g/dL.

The specificity of the panel outcomes in relation to relevant patient characteristics asked for a manageable format to disseminate the information. For that purpose, the panel recommendations are made available to practising physicians via an online tool that allows a quick ‘second opinion’ on the appropriateness of treatment for any given patient.

The applicability of the patient-specific recommendations in clinical practice is an important strength of the approach used in this study. However, we have to take into account that the panel recommendations are based on theoretical profiles; therefore, the validity of the online tool needs to be determined in prospective studies. In general, we feel that this tool may help increase the awareness of the importance of a timely diagnosis and appropriate treatment of iron deficiency in IBD.

## Conclusions

The RUAM was useful in establishing recommendations on the treatment of iron deficiency in patients with IBD. Iron supplementation was deemed necessary in almost all patients with either NAID or IDA. (High-dose) IV iron was more often considered appropriate than other options. The online tool may help disseminate the recommendations and increase awareness on the importance of treatment of iron deficiency in IBD.

## Authorship

*Guarantor of the article*: Walter Reinisch.

*Author contributions*: WR was the principal investigator, helped to obtain funding and chaired the panel meetings. WR and HS designed the study and prepared the draft manuscript. HS advised on the methodology, performed the statistical analyses and supervised the construction of the electronic tool. GW served the panel as a nonvoting expert in iron deficiency and anaemia. All other authors participated in the panel meetings, performed the appropriateness ratings, assisted in the interpretation of data and helped with the critical revision of the manuscript. All authors approved the final version of the manuscript.

## Appendix 1: Definitions used

### Non-anaemic iron deficiency (NAID)

- Biochemical evidence of iron deficiency and normal haemoglobin level
- Iron deficiency: ferritin level <30 ng/ml in inactive disease, <100 ng/ml in active disease
- Normal haemoglobin: men ≥ 13 g/dl, non-pregnant women ≥ 12 g/dl (WHO definition)

### Iron deficiency anaemia (IDA)

- Anaemia with biochemical evidence of iron deficiency
- Anaemia: men <13 g/dl, non-pregnant women <12 g/dl (WHO definition)
- Iron deficiency: ferritin level <30 ng/ml in inactive disease, <100 ng/ml in active disease

### (Previous) treatments

- Oral iron supplementation: optimised regimen according to the physician's judgment
- Low-dose IV iron supplementation (per single infusion): iron doses less than 500 mg
- High-dose IV iron supplementation (per single infusion): iron doses of 500 mg or more
- Previous treatment and outcome relate to a similar situation (profile)
- Insufficient response: reference point: serum ferritin <100 ng/ml, 6-8 weeks after treatment initiation
- Intolerance: unacceptable side effects
- Previous treatment with IV iron: in case of failure of IV iron it is assumed that oral treatment has also failed
- Blood transfusion: optimal regimen according to the physician's judgment

### Conditions associated with additional iron need

- For example: menstruation, history of anaemic episodes, previous gastrointestinal surgery

### Physical symptoms of iron depletion

- Symptoms (e.g. fatigue, shortness of breath) having a significant impact on quality of life.

### IBD activity status

- Definition active disease: evidence of inflammation based upon biochemical parameters, clinical symptoms and/or diagnostic imaging.

## SUPPORTING INFORMATION

Data S1 Literature overview in support of the European Panel Study on the appropriate management of iron deficiency in patients with inflammatory bowel disease.

## References

[b1] Gasche C, Berstad A, Befrits R (2007). Guidelines on the diagnosis and management of iron deficiency and anemia in inflammatory bowel diseases. Inflamm Bowel Dis.

[b2] Gisbert JP, Gomollón F (2008). Common misconceptions in the diagnosis and management of anemia in inflammatory bowel disease. Am J Gastroenterol.

[b3] Wells CW, Lewis S, Barton JR (2006). Effects of changes in hemoglobin level on quality of life and cognitive function in inflammatory bowel disease patients. Inflamm Bowel Dis.

[b4] Gasche C, Dejaco C, Waldhoer T (1997). Intravenous iron and erythropoietin for anemia associated with Crohn disease. A randomized, controlled trial. Ann Intern Med.

[b5] Gasche C (2000). Anemia in IBD: the overlooked villain. Inflamm Bowel Dis.

[b6] Gasche C, Lomer MC, Cavill I (2004). Iron, anaemia, and inflammatory bowel diseases. Gut.

[b7] Gisbert JP, Bermejo F, Pajares R (2009). Oral and intravenous iron treatment in inflammatory bowel disease: hematological response and quality of life improvement. Inflamm Bowel Dis.

[b8] Anker SD, Comin Colet J, Filippatos G (2009). Ferric carboxymaltose in patients with heart failure and iron deficiency. N Engl J Med.

[b9] Vaucher P, Druais PL, Waldvogel S (2012). Effect of iron supplementation on fatigue in nonanemic menstruating women with low ferritin: a randomized controlled trial. CMAJ.

[b10] Weiss G, Goodnough LT (2005). Anemia of chronic disease. N Engl J Med.

[b11] Theurl I, Aigner E, Theurl M (2009). Regulation of iron homeostasis in anemia of chronic disease and iron deficiency anemia: diagnostic and therapeutic implications. Blood.

[b12] Biancone L, Michetti P, Travis S (2008). European evidence-based Consensus on the management of ulcerative colitis: special situations. J Crohns Colitis.

[b13] Dignass A, Van Assche G, Lindsay JO (2010). The second European evidence-based Consensus on the diagnosis and management of Crohn's disease: current management. J Crohns Colitis.

[b14] Mowat C, Cole A, Windsor A (2011). Guidelines for the management of inflammatory bowel disease in adults. Gut.

[b15] Goddard AF, James MW, McIntyre AS (2011). Guidelines for the management of iron deficiency anaemia. Gut.

[b16] Muñoz M, Gómez-Ramírez S, García-Erce JA (2009). Intravenous iron in inflammatory bowel disease. World J Gastroenterol.

[b17] Stein J, Hartmann F, Dignass AU (2010). Diagnosis and management of iron deficiency anemia in patients with IBD. Nat Rev Gastroenterol Hepatol.

[b18] Brook RH, Chassin MR, Fink A (1986). A method for the detailed assessment of the appropriateness of medical technologies. Int J Technol Assess Health Care.

[b19] Fitch K, Bernstein SJ, Aguilar MS http://www.rand.org/pubs/monograph_reports/MR1269.

[b20] Shekelle P (2004). The appropriateness method. Med Decis Making.

[b21] Schusselé Filliettaz S, Juillerat P, Burnand B (2009). Appropriateness of colonoscopy in Europe (EPAGE II). Chronic diarrhea and known inflammatory bowel disease. Endoscopy.

[b22] Dubois RW (2009). On the second European panel on the appropriateness of Crohn's disease therapy (EPACT-II). J Crohns Colitis.

[b23] Caprilli R, Angelucci E, Cocco A (2005). Appropriateness of immunosuppressive drugs in inflammatory bowel diseases assessed by RAND method: Italian Group for IBD (IG-IBD) position statement. Dig Liver Dis.

[b24] Melmed GY, Spiegel BM, Bressler B (2010). The appropriateness of concomitant immunomodulators with anti-tumor necrosis factor agents for Crohn's disease: one size does not fit all. Clin Gastroenterol Hepatol.

[b25] Tricoci P, Allen JM, Kramer JM (2009). Scientific evidence underlying the ACC/AHA clinical practice guidelines. JAMA.

[b26] Shekelle PG, Kahan JP, Bernstein SJ (1998). The reproducibility of a method to identify the overuse and underuse of medical procedures. N Engl J Med.

[b27] Tobacman JK, Scott IU, Cyphert S (1999). Reproducibility of measures of overuse of cataract surgery by three physician panels. Med Care.

[b28] Quintana JM, Escobar A, Arostegui I (2006). Health-related quality of life and appropriateness of knee or hip joint replacement. Arch Intern Med.

[b29] Hemingway H, Crook AM, Feder G (2001). Underuse of coronary revascularization procedures in patients considered appropriate candidates for revascularization. N Engl J Med.

[b30] Reinisch W, Staun M, Bhandari S (2013). State of the iron: how to diagnose and efficiently treat iron deficiency anemia in inflammatory bowel disease?. J Crohns Colitis.

[b31] Ott C, Liebold A, Takses A (2012). High prevalence but insufficient treatment of iron-deficiency anemia in patients with inflammatory bowel disease: results of a population-based cohort. Gastroenterol Res Pract.

[b32] Voegtlin M, Vavricka SR, Schoepfer AM (2010). Prevalence of anaemia in inflammatory bowel disease in Switzerland: a cross-sectional study in patients from private practices and university hospitals. J Crohns Colitis.

[b33] Nemeth E, Tuttle MS, Powelson J (2004). Hepcidin regulates cellular iron efflux by binding to ferroportin and inducing its internalization. Science.

[b34] Evstatiev R, Marteau P, Iqbal T (2011). FERGIcor, a randomized controlled trial on ferric carboxymaltose for iron deficiency anemia in inflammatory bowel disease. Gastroenterology.

[b35] Bager P, Dahlerup JF (2010). The health care cost of intravenous iron treatment in IBD patients depends on the economic evaluation perspective. J Crohns Colitis.

[b36] Weiss G, Gasche C (2010). Pathogenesis and treatment of anemia in inflammatory bowel disease. Haematologica.

[b37] Bergamaschi G, Di Sabatino A, Albertini R (2010). Prevalence and pathogenesis of anemia in inflammatory bowel disease. Influence of anti-tumor necrosis factor-alpha treatment. Haematologica.

