# Supplementary material for: The management of iron deficiency in inflammatory bowel disease – an online tool developed by the RAND/UCLA appropriateness method
Source: Aliment Pharmacol Ther. 2013 Sep 17;38(9):1109–18. doi: 10.1111/apt.12493 (PMC3886296; doi:10.1111/apt.12493)
Supplement: Data S1 — Literature overview in support of the European Panel Study on the appropriate management of iron deficiency in patients with inflammatory bowel disease. [file apt0038-1109-sd1.pdf]

ismar healthcare
communication, education and decision support

Literature overview

in support of the European Panel Study on

The appropriate management of iron deficiency in patients with inflammatory bowel disease

Jessa Yperman, PhD
Herman Stoevelaar, PhD

Ismar Healthcare
Lier, Belgium

January 2012

Table of contents

1. INTRODUCTION 3
1.1. Aim of the overview 3
1.2. ID and anaemia in IBD 3
1.2.1 Prevalence and aetiology 3
1.2.2 Diagnosis 7
1.3. Treatment options for ID(A)/ACD in IBD patients 10
1.3.1. Iron supplementation 10
1.3.1.1. Oral iron supplementation 10
1.3.1.2. Intravenous/parenteral iron supplementation 10
1.3.2. Erythropoiesis-stimulating agents 15
1.3.3. Blood transfusion 15
2. METHODS 16
2.1. Search strategy 16
3. RESULTS 17
3.1. Evidence from clinical trials 17
3.1.1. Oral vs. oral iron supplementation 17
3.1.2 Oral vs. IV iron supplementation 18
3.1.3. IV vs. IV iron supplementation 25
3.1.4 Iron supplementation vs. iron supplementation + erythropoietic agent 27
3.2. Factors related to treatment choice 30
3.3. Guidelines and recommendations on treatment of ID 32
4. REFERENCES 37

1. INTRODUCTION

1.1. Aim of the overview

This literature overview was performed as part of the European RAND panel study on the appropriate management of iron deficiency (ID) in patients with inflammatory bowel disease (IBD). The RAND Appropriateness Method (RAM) aims at establishing appropriateness criteria at the patient-specific level by combining the best available evidence from clinical studies and the collective judgement of experts. An expert panel is asked to assess the appropriateness of particular therapeutic options for a large number of detailed hypothetical patient profiles. Where possible, judgements have to be based on evidence from clinical studies. If this information is lacking or is insufficiently detailed, experts may use their personal insights and experience as "complementary" evidence.
A literature overview forms a standard component of RAND panel studies and serves two purposes. Firstly, the results may be used to shape the research question and to determine the study design. To that aim, the results of an initial literature overview were discussed with the panel during the first meeting in Stockholm (October 2011). Secondly, an overview of available evidence from clinical studies may be supportive to panellists during the rating process. As such, this document –refined based upon the feedback of the panel during the first meeting- may be considered as a background document while performing the first round ratings.

1.2. ID and anaemia in IBD

1.2.1 Prevalence and aetiology

ID is a leading cause of anaemia, affecting over one-half billion people worldwide. At first sight, defining ID seems fairly simple: ID occurs if there is a deficit in total body iron, resulting from iron requirements that exceed the iron supply. However, there is no consensus on the right marker(s) to measure the deficit in total body iron in clinical practice, especially in patients with IBD [1]. In addition, the terms anaemia, ID, and ID anaemia (IDA) often are used interchangeably. The association between ID, IDA and anaemia is presented in Figure 1.

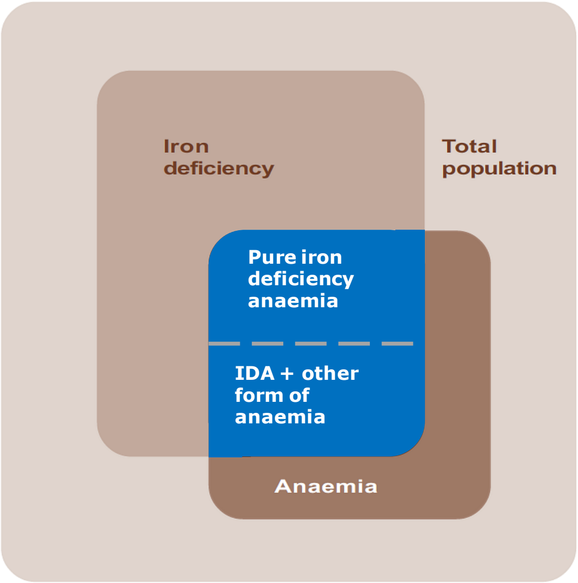

Figure 1: Schematic overview of the association between ID and anaemia (size is not representative of prevalence)
IDA: iron deficiency anaemia

According to a key paper of an international working group, the WHO definitions of anaemia (summarised in Table 1) apply to patients with IBD.

Table 1: Minimum haemoglobin and haematocrit levels used to define anaemia in people living at sea level according to WHO criteria [2]

Sex	Haemoglobin (Hb) threshold		Haematocrit threshold (%)
	g/dl	mmol/l	
Female			
Non-pregnant	12.0	7.45	36
Pregnant	11.0	6.83	33
Male	13.0	8.07	39

Because of the heterogeneity (in assessment, in the definitions used and in the populations studied), prevalence data on ID and IDA in IBD patients vary widely. In a 2008 paper from Gisbert and Gomollon, the mean weighted prevalence of ID in IBD calculated from the available studies was 45% [3]. Recent assessment of ID in IBD patients in the UK revealed a rate of 88% in children, 83% in adolescents and 55% in adults [4]. A Scandinavian study published in 2011, including 429 IBD outpatients, found ID in 35% of the patients [5].

The prevalence of anaemia in IBD also differs highly between studies, ranging from 6% to 74% as summarised in the Gisbert and Gomollon paper (Table 2) [3]. In a systematic review from 2004, prevalence of anaemia ranged from 8.8% to 73.7% (Table 3) [6].

back to Index

More recent studies found a prevalence of 65% in Italy [7], 40% in the UK [4] and 19% in Scandinavia [5]. In a Greek study published in 2011 including 100 IBD patients, the prevalence of anaemia was 41.2% for ulcerative colitis, 42.9% for Crohn's disease, whereas 30% of the IBD patients had IDA [8].

Table 2: Prevalence of anaemia in IBD patients [3]

Author	Number of patients	Population	Prevalence (%)
Bambach and Hill (11)	36	Outpatient	17
Beeken (12)	11	Outpatient	73
Beeken (13)	63	Inpatient	70
Burbige *et al.* (14)	58	Outpatient	52
Dyer *et al.* (15)	63	Inpatient	64
Ebinger *et al.* (16)	390	Outpatient	6
Ershler *et al.* (17)	7,200	Outpatient	13
Gasche *et al.* (18)	49	Outpatient	34
Greenstein *et al.* (19)	160	Inpatient	71
Harries *et al.* (20)	55	Outpatient	44
Hoffbrand *et al.* (21)	64	Inpatient and outpatient	44
Horina *et al.* (22)	85	Outpatient	33
Lakatos *et al.* (23)	254	Outpatient	60
Niv and Abukasis (24)	147	Outpatient	9
Niv *et al.* (25)	53	Outpatient	37
Oldenburg *et al.* (26)	–	Outpatient	29
Reilly *et al.* (27)	34	Inpatient	55
Revel-Vilk *et al.* (28)	63	Outpatient	41
Schreiber *et al.* (29)	676	Outpatient	31
Vijverman *et al.* (30)	170	Outpatient	25
Walker *et al.* (31)	2,894	Outpatient	13
Werlin and Grand (32)	19	Inpatient	74

Table 3: Prevalence of anaemia in IBD patients [6]

Population	Number of studies	Prevalence
Crohn's disease	9	10.2% - 72.7%
Ulcerative colitis	5	8.8% - 66.6%
Undifferentiated IBD	3	17.5% - 73.7%

IBD: inflammatory bowel disease

Aetiology

Although the definition of anaemia is simple, its underlying causes and mechanisms can be complex and overlapping (Figure 2) [9]. The major types of anaemia in IBD patients are IDA and anaemia of chronic disease (ACD). Besides these two, other factors like vitamin deficiencies and some commonly used IBD

drugs (such as 6 mercaptopurine, azathioprine, sulfasalazine and methotrexate) can aggravate anaemia in IBD a.o. by inhibiting erythropoiesis directly [10]. However, this is beyond the scope of this literature overview; therefore we refer to [11,9].

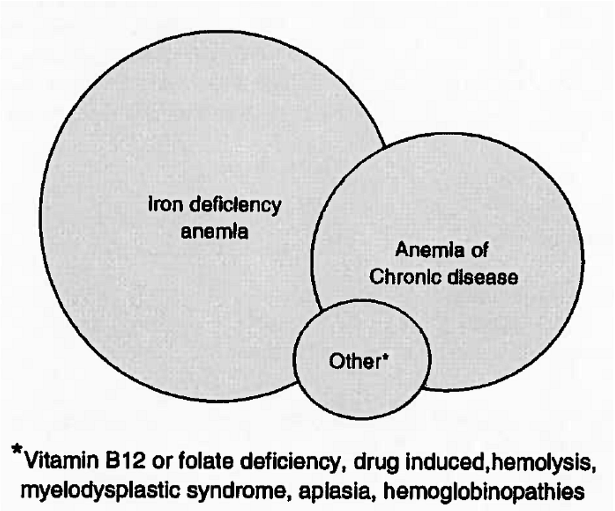

Figure 2: Aetiology of anaemia in IBD [1]

In IBD patients, chronic intestinal bleeding is a frequent cause of IDA (anaemia with biochemical evidence of ID) because the iron loss exceeds the amount of iron that can be absorbed from the diet resulting in a negative iron balance (absolute ID) [11]. Malnutrition with reduced iron intake and impaired iron uptake through the duodeno-jejunal mucosa can increase the severity of IDA (Figure 3) [2].
In active disease, inflammatory mediators may alter iron metabolism and cause ACD [12]. A number of mechanisms contribute to ACD such as retention of iron in the phagocytic system resulting in an inadequate iron supply to the bone marrow (functional ID), the negative impact of cytokins on proliferation and differentiation of erythroid progenitor cells and the impaired response to erythropoietin due to inflammation (Figure 3). This topic is extensively described in [13].

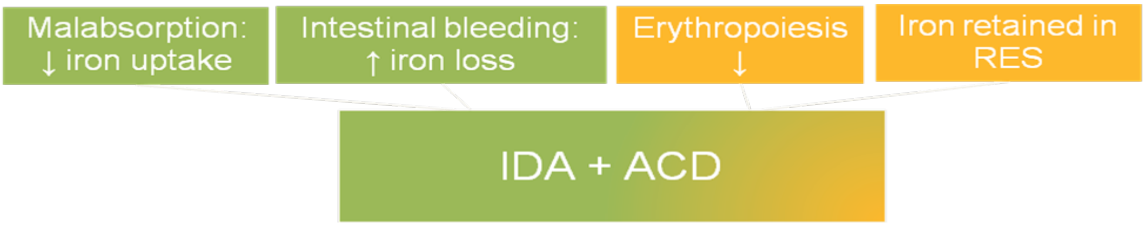

Figure 3: The origin of anaemia in IBD patients (typically a mix of IDA and ACD) is multifactorial [10]
ACD: anaemia of chronic disease; IDA: iron deficiency anaemia; RES: reticuloendothelial system

1.2.2 Diagnosis

Differentiation between IDA and ACD has therapeutic consequences: iron supplementation might be counterproductive in pure ACD, while it is necessary in case of IDA [12]. However, differentiating between both is difficult due to a lack of diagnostic markers that are accurate and easy to assess in daily clinical practice [1].

Assessment of the iron status in IBD is often difficult because inflammation influences parameters of the iron metabolism as illustrated in Table 4 [1,14]. Active disease directly affects standard measures of iron status, such as serum ferritin (indicator of storage iron content, acute phase reactant), serum iron, and soluble transferrin receptor (upregulated in case of functional ID) [15,16]. Therefore, the international working group suggested diagnostic criteria for ID depending on disease activity/level of inflammation (Table 5) [2].

Table 4: Laboratory findings in IDA, ACD and mixed IDA + ACD [16]

Laboratory measures	IDA	ACD	IDA + ACD
**Haemoglobin (Hb)**	↓	↓	↓
**Serum ferritin**	↓	↑	↑ or normal
Serum iron	↓	↓	↓
Transferrin	↑	↓ or normal	↓
**Transferrin saturation**	↓	↓	↓
Mean corpuscular volume	↓	↓ or normal	↓ or normal
Serum transferrin receptor (sTfR)	↑	↓ or normal	↑ or normal
sTfR-F index (sTfR:log ferritin)	High (>2)	Low (<1)	High (>2)
Reticulocyte Hb content (CHr) (pg)	<29	<29	<29
% hypochromic RBC	>5	>5	NA
Zinc protoporphyrin (µmol/mol haeme)	>40	>40	>40
Cytokine levels	Normal	↑	↑
**CRP**	Normal	↑	↑
Hepcidin	↓	↑	↑ or ↓

ACD: anaemia chronic disease; CRP: C-reactive protein; IDA: iron deficiency anaemia; NA: not applicable; RBC: red blood cells

Table 5: Degree of ID evaluated by serum ferritin and transferrin saturation [2]

**Degree of ID**	**Serum ferritin (µg/l)**	**Transferrin saturation (TfS) (%)**
Depleted iron stores in healthy adults or quiescent IBD	<30	<16
Depleted iron stores during active IBD	<100	<16
Adequate iron stores	>100	16-50
Potential iron overload	>800	>50

IBD: inflammatory bowel disease; ID: iron deficiency
Transferrin saturation (TfS) is the serum iron divided by the total iron-binding capacity (TIBC), which corresponds to circulating iron; it has a high sensitivity but a low specificity for detecting ID and has circadian fluctuations [16]

In addition, the ratio of the soluble transferrin receptor level to the log of the ferritin concentration (sTfR-F-index) could be helpful for differentiating IDA from ACD and mixed anaemia, but is not widely used in clinical practice [13,17,12].

An algorithm for differential diagnosis adapted from Weiss and Goodnough is given in Figure 4.

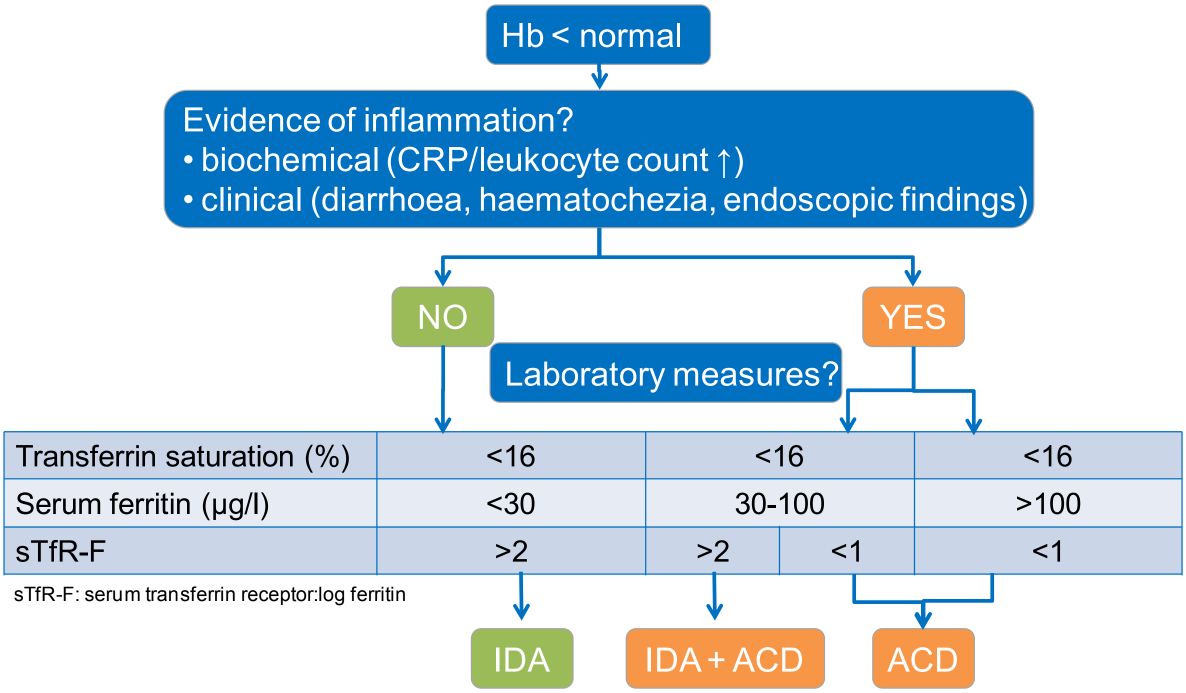

Figure 4: Algorithm for diagnostic differentiation between IDA, ACD and IDA + ACD, adapted from [13]

ACD: anaemia chronic disease; CRP: C-reactive protein; Hb: haemoglobin; IDA: iron deficiency anaemia; sTfR-F: serum transferrin receptor:log ferritin

Results from a study on differences in the regulation of iron homeostasis between IDA, ACD and IDA + ACD are presented in Figure 5 to illustrate that the search for accurate diagnostic markers that are easily applicable in clinical practice is complex.

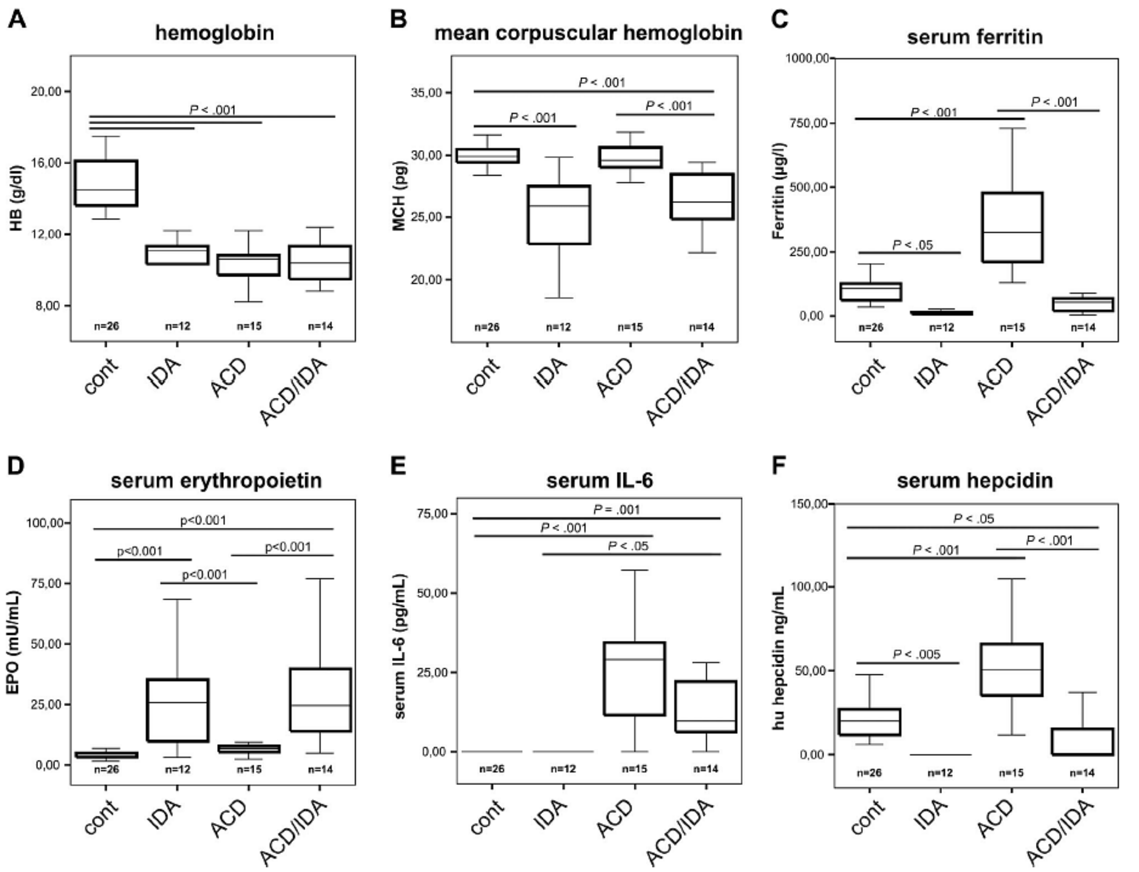

Figure 5: Laboratory parameters in control, IDA, ACD and IDA/ACD according to [16]

67 anaemic patients (not IBD patients) were categorised based upon their sTfR /log ferritin ratio

ACD: anaemia chronic disease; cont: control; EPO: erythropoietin; HB: haemoglobin; IDA: iron deficiency anaemia; IL: interleukin; MCH: mean corpuscular haemoglobin

According to the guidelines of the international working group, haemoglobin, serum ferritin, and C-reactive protein (CRP) should be used for laboratory screening of anaemia in IBD patients [2].

For a more comprehensive review of diagnosing ID/IDA and ACD in IBD patients we refer to [2,16,15,18].

back to Index

1.3. Treatment options for ID(A)/ACD in IBD patients

Although one out of three IBD patients has anaemia, it is an issue not addressed in many of them [11]. For a recent review on the optimal treatment for anaemia in patients with IBD, we refer to [44].

1.3.1. Iron supplementation

Iron replacement therapy cannot be comfortably undertaken until the cause of the iron deficit is ascertained. If iron supplementation is deemed appropriate, the Ganzoni formula is mostly used for dose calculation [19]:

$$\underset{[mg]}{\text{Total iron need}} = \underset{[kg]}{\text{Body weight}} \cdot (\text{Target Hb} - \underset{[g/dl]}{\text{Actual Hb}}) \cdot 2.4 + \underset{[mg]}{500}$$

1.3.1.1. Oral iron supplementation

The iron compound in the oral formations is most often ferrous (Fe^{2+}) iron (ferrous sulphate, ferrous fumarate, ferrous glucconate, carbonyl iron), although the ferric (Fe^{3+}) polysaccharide-iron complex is also available. Numerous formulations exist such as tablets, capsules, liquid filled capsules, coated tablets, chewable tablets, liquids, combination products and extended-release products. The coated formulations are usually better tolerated but less absorbed.
Common adverse events (AEs) of oral iron supplements include nausea, epigastric discomfort, and constipation, all of which are dose-related. In patients with IBD, the use of oral iron supplementation is further limited by reduced absorption in the setting of inflammation and gastrointestinal side effects leading occasionally to exacerbation of the inflammatory process and disease activation, since 90% of ingested iron is not absorbed, and therefore can cause oxidative tissue damage [20,21,3].
Restoring iron stores with oral iron supplementation usually takes 3 to 4 months. The main factor in favour of oral iron supplementation is convenience [3].

1.3.1.2. Intravenous/parenteral iron supplementation

Intravenous (IV) iron supplementation was already introduced several decades ago. The first formulations were quite toxic and a test dose was necessary when using the first dextran-containing IV iron preparations because of the risk of anaphylaxis. Recently, newer IV iron formulations have appeared on the market, which do not contain a requirement for a test dose. For some of these formulations a much higher dose of iron can be delivered as a single

back to Index

administration with acceptable safety and without significant AEs. The most common AEs currently associated with IV iron supplementation are nausea, abdominal pain, constipation, diarrhoea, injection site reactions, metallic taste, headache, dizziness, rash with an incidence of 1-3% [22].
The long-term safety of the newer IV iron formulations in IBD patients is not yet established; numbers of non-chronic kidney disease patients receiving these formulations are not large enough to draw definitive conclusions. We therefore refer to a review on the safety of IV iron agents (iron sucrose, iron gluconate, low molecular weight iron dextran (LMWID) and high molecular weight iron dextran (HMWID) in chronic kidney disease patients [23].

An overview of the available IV formulations, a.o. based upon the summary of product characteristics (SPCs) is given in Table 6. SPCs of the marketed products are available online:

Dexferrum:
http://www.americanregent.com/documents/Product16PrescribingInformation.pdf

Cosmofer: http://emc.medicines.org.uk/medicine/14139/SPC/CosmoFer/

Ferrlecit: http://products.sanofi-aventis.us/ferrlecit/ferrlecit.html

Venofer:
http://www.medicines.org.uk/EMC/medicine/24168/SPC/Venofer+(iron+sucrose)/

Ferinject:
http://www.medicines.org.uk/EMC/medicine/24167/SPC/Ferinject+(ferric+carboxymaltose)/

Feraheme:
http://www.feraheme.com/Feraheme%20Label_FDA_approved_version%20_%20June_2011.pdf

Monofer:
http://www.medicines.org.uk/emc/medicine/23669/SPC/monofer%20100mg~ml%20solution%20for%20injection~infusion/

Table 6: Overview of IV iron supplementation formulations [24,25,26,22,2,16,23]

	HMW iron dextran	LMW iron dextran	Iron gluconate	Iron sucrose	Ferric carboxymaltose	Ferumoxytol	Iron isomaltoside 1000
**Trade name (Europe)**	Dexferrum	Cosmofer	Ferrlecit	Venofer	Ferinject	Feraheme	Monofer
**Manufacturer**	Luitpold Pharmaceuticals	Pharmacosmos	Sanofi-Aventis	Vifor Int.	Vifor Int.	AMAG Pharmaceuticals	Pharmacosmos
**Chemical properties**							
**Carbohydrate**	dextran (branched polysaccharide)	dextran (branched polysaccharide)	gluconate (monosaccharide)	sucrose (disaccharide)	carboxymaltose (branched polysaccharide)	carboxymethyl-dextran (branched polysaccharide)	isomaltoside 1000 (unbranched linear oligosaccharide)
**MW (kD)**	265	73-165	<50	30-100	150	750	1000
**Complex type**		type I	type III	type II	type I		
**Complex stability**	robust, strong	robust, strong	labile, weak	semi-robust, moderately strong	robust, strong	robust, strong	
**Acute toxicity due to labile iron release**	low	low	high	medium	low	low	low
**Plasma half-life (h)**		20 (total iron)	1	6	7-12	15	20 (total iron)
**Direct iron donation to transferring (% injected dose)**		1-2	5-6	4-5	1-2		

	HMW iron dextran	LMW iron dextran	Iron gluconate	Iron sucrose	Ferric carboxy-maltose	Ferumoxytol	Iron isomaltoside 1000
**Trade name (Europe)**	Dexferrum	Cosmofer	Ferrlecit	Venofer	Ferinject	Feraheme	Monofer
**Manufacturer**	Luitpold Pharmaceuticals	Pharma-cosmos	Sanofi-Aventis	Vifor	Vifor	AMAG Pharma-ceuticals	Pharmacosmos
**Dosing**							
**Iron concentration**	50 mg/ml	50 mg/ml	12.5 mg/ml	20 mg/ml	50 mg/ml	30 mg/ml	100 mg/ml
**Intravenous drip infusion**							
**Max. single dose**	-	200 mg	125 mg	200 mg	15 mg/kg (1000 mg)	-	20 mg/kg
**Rate of administration**	-	41.25 min	60 min	100 mg: 15 min<br>200 mg: 30 min	<500 mg: 6 min<br>500-1000 mg: 15 min	-	0-5 mg/kg: 15 min<br>6-10 mg/kg: 30 min<br>11-20 mg/kg: 60 min
**One dose iron repletion (total dose infusion)**							
**One dose iron repletion (TDI)**	no	20 mg/kg	no	no	no	no	20 mg/kg
**Rate of administration**	-	240-360 min	-	-	-	-	0-10 mg/kg: 30 min<br>11-20 mg/kg: 60 min
**Intravenous bolus injection**							
**Max. injectable single dose**	100 mg	200 mg	125 mg	200 mg	200 mg	510 mg	100-200 mg
**Rate of administration**	2 min	35 min (including waiting time after test dose)	10 min	10 min	bolus push	17 s	2-4 min
**Test dose required**	yes	yes (wait 15 min after injection)	no	yes (except US/UK)	no	no, but must wait 30 min after injection	no

	HMW iron dextran	LMW iron dextran	Iron gluconate	Iron sucrose	Ferric carboxymaltose	Ferumoxytol	Iron isomaltoside 1000
**Trade name (Europe)**	Dexferrum	Cosmofer	Ferrlecit	Venofer	Ferinject	Feraheme	Monofer
**Manufacturer**	Luitpold Pharmaceuticals	Pharmacosmos	Sanofi-Aventis	Vifor Int.	Vifor Int.	AMAG Pharmaceuticals	Pharma-cosmos
**Safety profile**							
**Acute toxicity due to labile iron release**	low	low	high	medium	low	low	low
**Risk of dextran-induced anaphylaxis**	yes	yes	no	no	no		
**Relative risk of SAEs**	high	moderate	low	very low	n.a.	very low	
**Risk of life-threatening AEs* [23]**	$11.3/10^6$ doses	$3.3/10^6$ doses	$0.9/10^6$ doses	$0.6/10^6$ doses	n.a.		
**Risk of death* [23]**	$0.78/10^6$ doses	$0.75/10^6$ doses	$0.25/10^6$ doses	$0.11/10^6$ doses	n.a.		
**Pregnancy category**	C	C	B	B	n.a.	C	

*Data from chronic kidney disease patients
HMW: heigh molecular weight; LMW: low molecular weight; MW: molecular weight; n.a.: not applicable

back to Index

1.3.2. Erythropoiesis-stimulating agents

Since erythropoiesis is compromised in patients with ACD, erythropoiesis-stimulating agents (ESAs) can be used to correct this type of anaemia. However, ESAs only complete the range of available therapeutic options and are not generally seen as first-line treatment [27]. Treatment for ACD should start with controlling inflammation and IV iron therapy [27].
Iron supplementation is necessary during ESA therapy because of the increased iron demand due to enhanced erythropoiesis [3,2].

The three ESAs routinely available in clinical practice are epoetin alpha and epoetin beta (both formulations of recombinant human erythropoietin) and darbepoietin alfa [20]. The most common side effect is hypertension. The development of anti-erythropoietin antibodies is a very rare but serious AE; however, overall ESAs have an excellent safety profile [20,27].

1.3.3. Blood transfusion

According to a circular of AABB, the American Red Cross, America's Blood Centers, and the Armed Services Blood Program, red-cell-containing components should not be used to treat anaemia that can be corrected with specific medications such as iron, vitamin B12, folic acid, or erythropoietin [28]. This is in accordance with the guidelines of the international working group stating that blood transfusion is no substitute for the treatment of IDA with IV iron, possibly in combination with ESA [2].
The incidence of AEs is much smaller for IV iron supplementation than for blood transfusion (Table 7) [22].

Table 7: Safety outcomes of IV iron supplementation compared to blood transfusion [22]

**Intervention**	**Risk of life-threatening AEs**	**Risk of deaths**
IV iron (CKD) [23]	$2.2/10^6$ doses	$0.4/10^6$ doses
Allogeneic blood transfusion	$10.0/10^6$ doses	$4.0/10^6$ doses

AEs: adverse events; CKD: chronic kidney disease; IV: intravenous

Therefore, timely recognition and appropriate treatment of anaemia should reduce the need for blood transfusion.
Furthermore, blood transfusions do not correct the underlying pathology of anaemia and do not have a lasting effect and therefore should only be used in case of life-threatening anaemia [10]. Even if the haemoglobin is corrected by transfusion, iron supplementation is still necessary [2].

2. METHODS

This report provides an overview of current clinical evidence on different treatment options for ID/IDA in IBD patients. It focuses on efficacy and safety, factors related to treatment choice and guideline recommendations. Relevant published literature was screened and priority was given to publications with the highest level of evidence: meta-analyses and randomised controlled trials (RCTs). Cost-effectiveness studies and economical evaluations were not taken into account since cost issues will not be considered in this RAND study.

2.1. Search strategy

In PubMed, the search for retrieving the appropriate references was performed by combining the medical subject heading (MeSH) terms: inflammatory bowel diseases' AND 'anemia, iron-deficiency', without limits. In addition the following search using ('anemia/drug therapy' (MeSH) OR 'anemia/therapy' (MeSH)) AND 'inflammatory bowel diseases' (MeSH) with limits: humans, clinical trial, practice guideline, meta-analysis, RCT, review was performed. Only published full English-language papers were included. Bibliographies of retrieved papers were also screened for additional references. The initial literature search was performed in June 2011. Articles retrieved during an additional search at the end of December 2011 were also included.

3. RESULTS

3.1. Evidence from clinical trials

Our literature search did not retrieve any meta-analysis in the field of IDA in IBD patients. Eight RCTs were found in addition to five comparative non-RCTs (Table 8).

Table 8: Overview of RCTs and comparative studies of iron supplementation in patients with IBD

	Oral iron	IV iron
Oral iron	1 RCT	
IV iron	4 RCTs<br>3 non-RCTs	1 RCT
Iron + ESA	1 RCT	1 RCT<br>2 non-RCTs

ESA: erythropoiesis-stimulating agent; IV: intravenous; RCT: randomised controlled trial

The most important efficacy outcome is treatment response (haemoglobin increase). Considering safety/tolerability we focused on discontinuation due to AEs/poor tolerability.

3.1.1. Oral vs. oral iron supplementation

There is only one RCT comparing two oral iron compounds in patients with IBD [29] and we could not find comparative non-RCTs on this topic.

In the non-blinded, Norwegian RCT, 41 patients with IBD were randomised to ferrous sulphate or iron poly-maltose complex for 2 weeks to evaluate oxidative tissue damage and clinical disease activity. Oral ferrous iron supplements are poorly absorbed and may reinforce intestinal tissue injury by catalysing production of reactive oxygen species. The iron-polymaltose complex contains iron in a non-ionic form, making it less toxic. Two markers of oxidative tissue damage were evaluated: plasma malondialdehyde (MDA) and urine 8-iso prostaglandin F 2 alpha (8-iso-$PGF_{2\alpha}$) (Figure 6). There was no correlation between markers of oxidative stress and clinical disease activity. Clinical disease activity was unchanged after both treatments. The duration of treatment was too short to be a study of efficacy on correction of ID.

In the ferrous sulphate group 3/21 (14.3%) patients discontinued due to an intolerable increase in stool frequency, abdominal pain and nausea, while 1/20 (5%) patients discontinued in the iron-polymaltose group. Increased nausea occurred in nine patients on ferrous sulphate and seven on iron polymaltose.

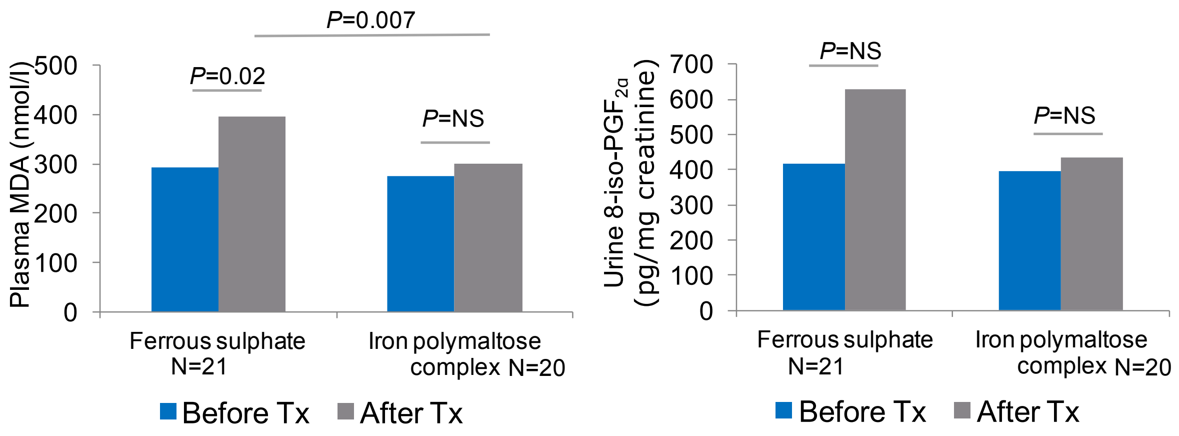

Figure 6: Ferrous sulphate treatment significantly increased plasma MDA and tended to increase 8-iso-PGF$_{2\alpha}$; these changes were not found for iron-polymaltose treatment [29]

8-iso-PFG$_{2\alpha}$: 8-iso prostaglandin F 2 alpha; MDA: malondialdehyde; NS: not significant; Tx: therapy

3.1.2 Oral vs. IV iron supplementation

Our literature search retrieved four RCTs (Table 9) and three comparative non-RCTs (Table 10) comparing oral iron with IV iron supplementation.

A review on this topic was published in 2009, including 669 patients from three RCTs, one of the comparative non-RCTs and three non-comparative studies [22]. The main findings were that despite differences in baseline [haemoglobin], iron dose and follow-up, there was a higher response rate on IV iron compared to oral iron (weighted mean 74% vs. 65%), especially in the RCTs (weighted mean 72% vs. 58%). In addition, the rate of treatment discontinuation due to AEs was lower with IV iron compared to oral iron.

The first RCT is a small 14-day study and therefore too short to evaluate correction of anaemia [30]. Oral iron did increase clinical disease activity

(assessed by Harvey-Bradshaw Simple Index in Crohn's disease patients and Simple Clinical Colitis Activity Index in ulcerative colitis patients), while IV iron supplementation did not. Therefore, it is concluded that oral iron should be given with caution to patients with IBD.

The second RCT, published in 2005 and including 46 patients with baseline [haemoglobin] ≤10.5 g/dl (F) or ≤11.0 g/dl (M) and TfS ≤20% and/or serum ferritin ≤20 µg/l showed that short-term efficacy as measured by change in [haemoglobin] was similar between both groups (Table 9) [31]. Disease activity as determined by Crohn's Disease Activity Index (CDAI) and Colitis Activity Index (CAI) improved to the same extent in both groups. However, an increase in serum [ferritin] was only observed in the IV iron group (Figure 7). Although the total number of AEs was comparable between both groups, the AE profile was quite different: in the oral iron group 71% of AEs were gastrointestinal AEs, while this was only 10% in the IV iron group.
It has to be noted that this study was underpowered.

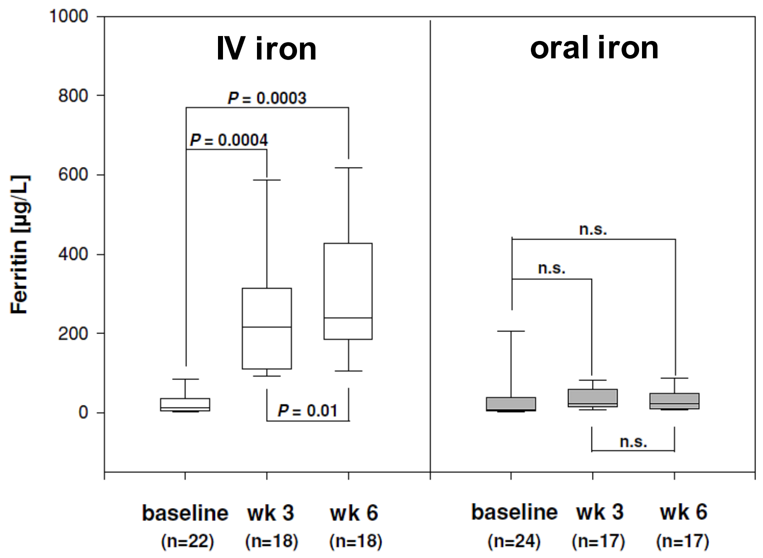

Figure 7: Changes in serum [ferritin] in response to IV iron or oral iron [31]
Boxes represent median and interquartile range, error bars show 10th and 90th percentiles
n.s.: not significant

The third RCT, FERINJECT, is a non-inferiority trial in which patients with baseline [haemoglobin] ≤11.0 g/dl and TfS <20% or serum [ferritin] <100 µg/l were randomised 2:1 to IV ferric carboxymaltose or oral ferrous sulphate (Table 9) [32]. Based on the median improvement in [haemoglobin] IV iron was non inferior to oral iron in this trial. Response on oral iron was slower than on IV iron as well with respect to haemoglobin, as to serum ferritin and transferrin saturation (Figure 8).

AEs were experienced in 42.9% of the patients on oral iron and 56.9% of patients in the IV iron group. Numbers for treatment-related AEs are given in Table 9. Abdominal complaints were more frequently observed with oral iron. The number of patients on oral iron who discontinued treatment due to AE was unexpectedly low (7.9%); oral iron intolerance is expected to occur in at least 25% of IBD patients. Since patients with a history of iron intolerance were excluded, selection bias for iron-intolerant patients may explain this finding.

The most recent RCT, a Swedish trial including 91 IBD patients with IDA, not in active relapsing state of IBD with a baseline [haemoglobin] ≤11.5 g/dl and serum ferritin <300 µg/l and ID defined by S-iron, transferrin and TfS showed that IV iron was superior to oral iron in correcting [haemoglobin] and iron stores (Table 9 and Figure 9) [33]. Disease activity indices were low at the start of the study and remained largely unchanged in both treatment groups.
Only 48% of the patients in the oral group tolerated the prescribed dose. Adverse reactions seen on oral iron were dominated by gastrointestinal symptoms.

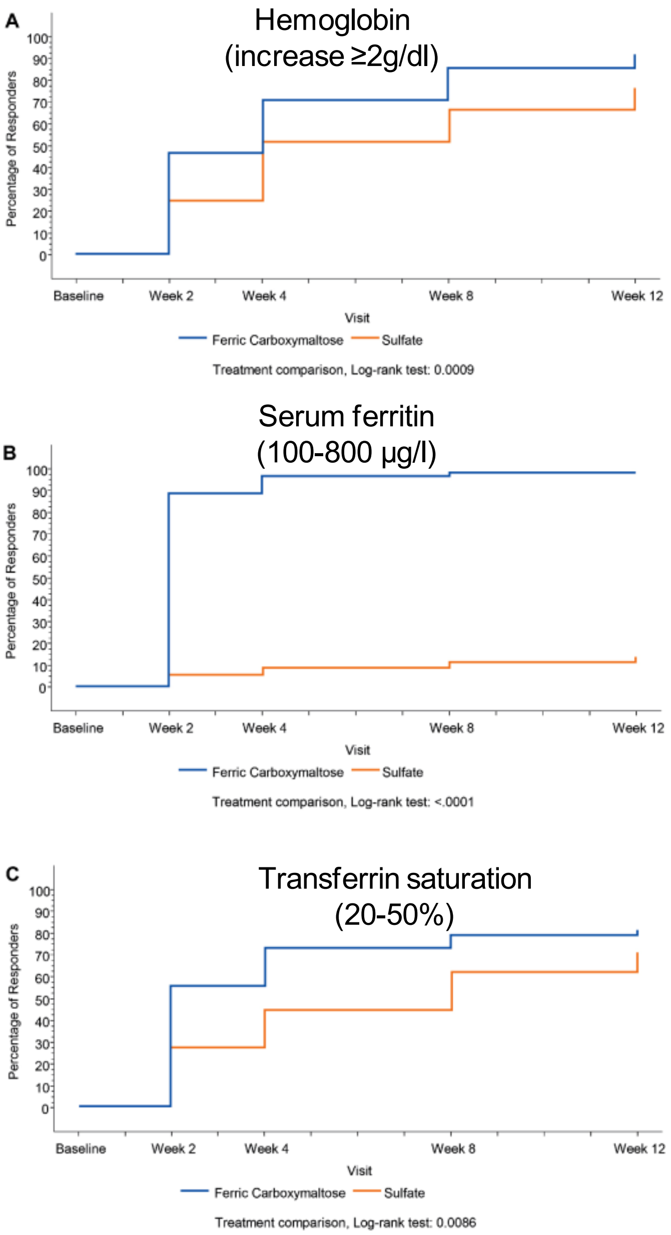

Figure 8: Response rate of [haemoglobin], serum ferritin and transferrin saturation was higher in the IV iron group than in the oral iron group [32]

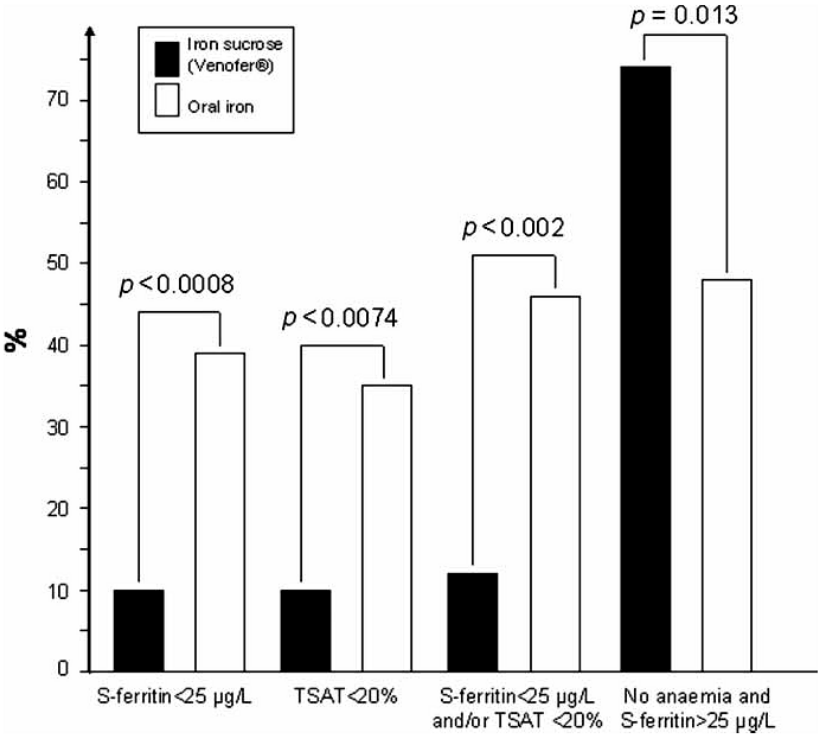

Figure 9: Normalisation of serum ferritin and transferrin saturation occurred more in patients on IV iron [33]
Vertical axis= proportion of patients
TSAT: transferrin saturation

Table 9: Overview of RCTs comparing oral iron to IV iron supplementation
only published full English-language papers (1980-present), no abstracts

Oral vs. IV: RCTs									
**Study**	**Design**	**Patients**	**Treatment**	**Mean total iron dose**	**N**	**Response**	**Hb: baseline-after therapy (g/dl)**	**AEs (% of patients)**	**AEs leading to discontinuation (% of patients)**
Erichesen 2005 [30]	randomised non-blinded cross-over (14 d)	IBD + IDA	oral ferrous fumarate<br><br>IV iron sucrose	1680 mg (120 mg/d)<br><br>600 mg (3x 200 mg)	19	NR<br><br>NR	11.6-11.8<br><br>10.6-11.3§<br>*P*<0.05	2 (10.5%)<br><br>6 (31.6%)	2 (10.5%)<br><br>0
Schröder 2005 [31]	open-label, multi-centre RCT (6 w)	IBD + IDA	oral ferrous sulphate<br><br>IV iron sucrose	median 4200 mg (100-200 mg/d)<br><br>median 1418 mg (initial dose 7 mg/kg + 5x 200 mg)	24<br><br>22	53%*<br><br>55%<br><br>*P*=0.85	mean: 9.6-11.7<br><br>9.8-12.3	21 (41.7%)<br><br>20 (50.0%)	5 (20.8%)<br><br>1 (4.5%)<br><br>*P*=0.19
Kulnigg 2008 [32] FERINJECT	open-label, multi-centre RCT (12 w)	IBD + IDA	oral ferrous sulphate<br><br>IV ferric carboxymaltose	16800 mg (200 mg/d)<br><br>1-3 infusions of 500-1000 mg (max. 1000 mg/w)	63<br><br>137	68.3%*<br><br>76.5%<br><br>*P*=0.0009$	median: 9.1-12.1<br><br>8.7-12.3<br><br>*P*=0.70	14 (22.2%)<br><br>39 (28.5%)	5 (7.9%)<br><br>2 (1.5%)<br><br>*P*=0.057
Lindgren 2009 [33]	investigator-blinded, multi-centre RCT (20 w)	IBD + IDA	oral ferrous sulphate<br><br>IV iron sucrose	38387 mg (200-400 mg/d)<br><br>1708 mg (200 mg/1-2 w)	46<br><br>45	47%*<br><br>66%<br><br>*P*=0.07	mean: 10.4-NR<br><br>10.5-NR	NR<br><br>NR	11 (24%)<br><br>1 (2.2%)

*response: Hb increase ≥2.0 g/dl

§ comparison before-after treatment (not comparison between treatment groups)

$cumulative Hb responder rate over 12 weeks; Kaplan-Meier analysis

AEs: adverse events; d: day; Hb: haemoglobin; IBD: inflammatory bowel disease; IDA: iron deficiency anaemia; IV: intravenous; NR: not reported; RCT: randomised controlled trial; w: weeks

Table 10: Overview of comparative non-RCTs on oral iron vs. IV iron supplementation

Oral vs. IV: comparative studies									
**Study**	**Design**	**Patients**	**Treatment**	**Mean total iron dose**	**N**	**Response**	**Mean Hb: baseline-after therapy (g/dl)**	**AEs (% of patients)**	**AEs leading to discontinuation (% of patients)**
Wells 2006 [34]	open-label (6 mo)	IBD + anaemia in preceding 12 mo	no treatment (if no anaemia at baseline)	none	29	3.4%*	no treatment: 13.2-13.4	NR	NR
			oral ferrous sulphate	3x 200 mg/d	12	33.3%	oral + IV iron: 11.3-13.5	NR	NR
			IV iron sucrose (irresponsive or intolerant to oral iron or Hb <10.5 ng/dl)	1400 mg (2x 200 mg/w)	9	55.6%		0	0
Gisbert 2009 [35]	open-label multi-centre (6 mo)	IBD + IDA	oral ferrous sulphate if Hb >10g/dl	NR (106 mg/d)	78	89%**	10.8-NR	NR	4 (5.1%)
			IV iron sucrose if Hb <10 g/dl	NR(2x 200 mg/w)	22	77%	8.8-NR	0	0
Khalil 2011 [36]	retrospective case-matched (±8 w)	IBD + IDA	oral ferrous sulphate (25), ferrous fumarate (3), polysaccharide-iron complex (2), sodium feredetate (2), ferrous gluconate (1)	103 mg elemental iron (6.8 mg/d)	33	39%**	11.3-11.9	NR	5/33 (15%)
			IV iron dextran	949 mg	33	30%<br>*P*=0.61	9.3-11.3<br>*P*<0.0001	NR	2/35 (6%) anaphylactoid reaction to test dose

*response: Hb increase ≥2.0 g/dl

**response: complete Hb normalisation (male: ≥13 g/dl; female: ≥12 g/dl)

AEs: adverse events; d: day; Hb: haemoglobin; IBD: inflammatory bowel disease; IDA: iron deficiency anaemia; IV: intravenous; mo: months; NR: not reported; w: weeks

3.1.3. IV vs. IV iron supplementation

The only RCT comparing two IV iron formulations in patients with IBD is the non-inferiority RCT FERGIcor including 483 IBD patients from 14 European countries with IDA (inclusion criteria: haemoglobin ≤12.0 g/dl (F) and ≤13.0 g/dl (M) and serum ferritin <100 µg/l)and mild-moderate or quiescent IBD (CDAI <220 or CAI ≤7) [37]. Further study characteristics are shown in Table 11.

The iron dosing regimen was calculated differently for patients receiving IV iron sucrose (individually by Ganzoni formula: total iron dose: [body weight x (target haemoglobin - actual haemoglobin)] x2.4 + iron storage depot) than for patients receiving IV ferric carboxymaltose (FCM) (fixed dose based on haemoglobin and weight above or below 67 kg) resulting in a higher mean total iron dose in the FCM group (Table 11).

The response rate was significantly better in the FCM group (Table 11). In addition at the end of the 12-week treatment period more patients on FCM had a normal haemoglobin (72.8% vs. 61.8% on iron sucrose; P=0.015) and a serum ferritin level ≥100 µg/l (42.5% vs. 27.3%; P=0.001). One treatment-related serious AE (pulmonary embolism) was reported in the FCM group.

Table 11: Overview of RCTs comparing IV to IV iron supplementation; only published full English-language papers (1980-present), no abstracts

RCT comparing IV iron preparations									
**Study**	**Design**	**Patients**	**Treatment**	**Mean total iron dose**	**N**	**Response**	**Hb: baseline-after therapy (g/dl)**	**AEs (% of patients)**	**AEs leading to discontinuation (% of patients)**
Evstatiev 2011 [37] FERGIcor	open-label, multi-centre RCT (12 w)	mild to moderate or quiescent IBD + IDA	IV ferric carboxymaltose	1377 mg (max. 3x 500-1000 mg)	244	65.8%*	10.1-NR	34 (13.9%)	7 (2.9%)
			IV iron sucrose	1160 mg (max. 11x 200 mg)	239	53.6%	10.3-NR	27 (11.3%)	2 (0.8%)
						P=0.004		*P*=0.413	*P*=0.176

*response: Hb increase ≥2.0 g/dl

AEs: adverse events; Hb: haemoglobin; IBD: inflammatory bowel disease; IDA: iron deficiency anaemia; IV: intravenous; mo: months; NR: not reported; RCT: randomised controlled trial; w: weeks

3.1.4 Iron supplementation vs. iron supplementation + erythropoietic agent

We could retrieve two rather small RCTs (Table 12) and two other comparative studies (Table 13) in which iron supplementation alone was compared to iron supplementation + ESA in patients with IBD.
Both RCTs demonstrated the efficacy of ESA in patients unresponsive to iron supplementation.

In the first RCT patients with severe anaemia despite 6 weeks of oral iron, supplementation were randomised to further oral iron therapy + placebo or oral iron + ESA [38]. (Patients with severe ID based on ferritin level were excluded). While the mean haemoglobin level decreased over the 12-week study period in the iron monotherapy group, it increased when combined with ESA (Figure 10A).

In the second RCT, anaemic Crohn's disease patients who were unresponsive to 2 months oral iron supplementation or could not tolerate oral iron received IV iron sucrose with or without ESA. IV iron caused a considerable haemoglobin increase that was faster and larger when combined with ESA (Figure 10B) [39].

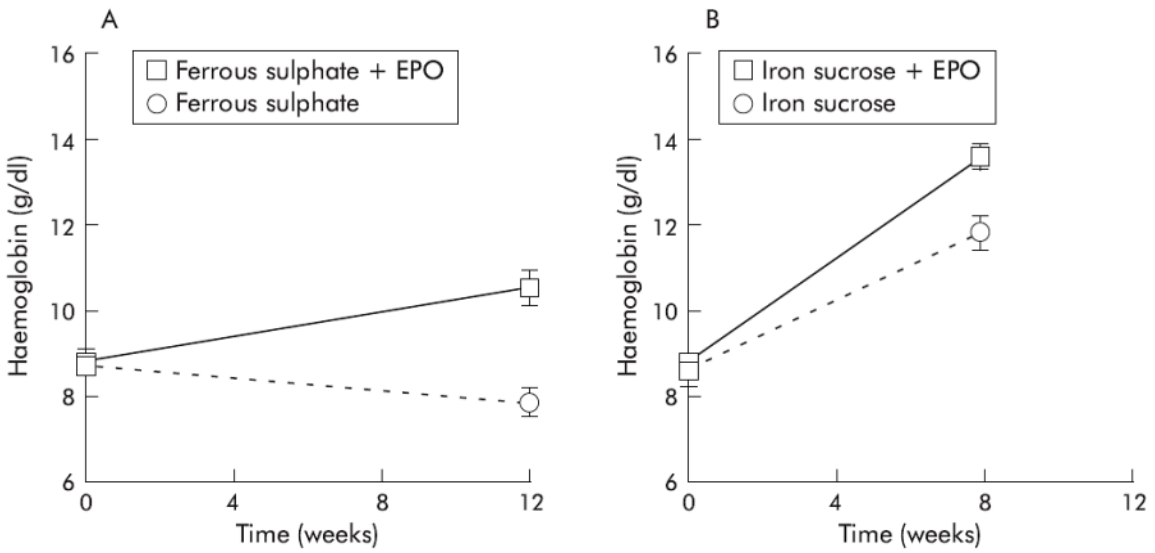

Figure 10: Haemoglobin level over time while on oral iron supplementation alone or with ESA (A) or on IV iron supplementation alone or with ESA (B)
[11]
EPO: erythropoietin

Table 12: Overview of RCTs comparing iron to iron supplementation + ESA; only published full English-language papers (1980-present), no abstracts

Iron (IV or oral) vs. iron + ESA: RCTs									
**Study**	**Design**	**Patients**	**Treatment**	**Mean total iron dose**	**N**	**Response**	**Mean Hb: baseline-after therapy (g/dl)**	**AEs (% of patients)**	**AEs leading to discontinuation (% of patients)**
Schreiber 1996 [38]	double-blind placebo-controlled RCT (12 w)	IBD + Hb ≤10.0 g/dl despite 6 w of oral iron	oral ferrous sulphate + placebo	100 mg/day	17	24%***	8.7-7.8		
			oral ferrous sulphate + rHuEPO (epoetin alpha)	100 mg/day + 2x 150 IU/kg body weight/w	17	82%<br>*P*=0.002	8.8-10.5<br>*P*<0.001		
Gasché 1997 [39]	double-blind placebo-controlled RCT (8 w)	CD + Hb ≤10.5 g/dl; unresponsive or intolerant to oral iron	IV iron sucrose + placebo	2000 mg (2x 200 mg/w for 2 w; 1x 200 mg/w for 6 w)	20	75%*	8.5-11.8	NR	0
			IV iron sucrose + rHuEPO (epoetin alpha)	2000 mg + 3600 IU/kg body weight (3x 150 IU/kg body weight/w)	19	95%<br>*P*=0.20<br>cumulative response: *P*=0.036	8.7-13.6<br>*P*=0.004	NR	0
	open-label phase (8 w)	non-responders in double-blind phase	IV iron sucrose + rHuEPO (epoetin alpha)	1600 mg (1x 200 mg/w) + 3600 IU/kg body weight (3x 150 IU/kg body weight/w)	5	100%	NR (mean increase 3.6)	NR	0
				1600 mg (1x 200 mg/w) + 7200 IU/kg body weight (3x 300 IU/kg body weight/w)	1	100%	NR (mean increase 3.6)	NR	0

*response: Hb increase ≥2.0 g/dl

***response: Hb increase >1.0 g/dl

AEs: adverse events; CD: Crohn's disease; d: day; Hb: haemoglobin; IBD: inflammatory bowel disease; IU: international units; IV: intravenous; NR: not reported; RCT: randomised controlled trial; w: weeks

Table 13: Overview of comparative non-RCTs on iron vs. iron supplementation + ESA; only published full English-language papers (1980-present), no abstracts

Iron (IV or oral) vs. iron + ESA: comparative non-RCTs									
**Study**	**Design**	**Patients**	**Treatment**	**Mean total iron dose**	**N**	**Response**	**Mean Hb: baseline-after therapy (g/dl)**	**AEs (% of patients)**	**AEs leading to discontinuation (% of patients)**
Gasché 1994 [40]	open-label (5 w)	CD + Hb ≤10.5 g/dl	IV iron sucrose	1000 mg (5x 200 mg)	2	NR	9.5-11.5	0	0
			IV iron sucrose + rHuEPO	1000 mg (5x 200 mg) + rHuEPO (3x 150 IU/kg body weight/w)	2		9.1-14.4	0	0
Gasche 1999 [41]	single-centre open-label: phase 1 (8 w)	UC + Hb ≤10.5 g/dl; unresponsive or intolerant to oral iron	IV iron sucrose	2000 mg (2x 200 mg/w for 2 w; 1x 200 mg/w for 6 w)	20	80%*	8.3-11.9§ *P*<0.001	7 (35%) due to iv iron	0
	phase 2 (8 w)	non-responders in phase 1	IV iron sucrose + rHuEPO (epoetin alpha)	1600 mg (1x 200 mg/w) + 3600 IU/kg body weight (3x 150 IU/kg body weight/w)	3	66.7%	9.5-12.8	0 due to rHuEPO	0

*response: Hb increase ≥2.0 g/dl

§comparison before-after treatment (not comparison between treatment groups)

AEs: adverse events; CD: Crohn's disease; Hb: haemoglobin; IU: international units; IV: intravenous; NR: not reported; UC: ulcerative colitis; w: weeks

3.2. Factors related to treatment choice

We could retrieve three studies (two full publications, one abstract) with data exploring factors predicting response to IV iron. Data are summarised in Table 14.
The outcomes of these three studies are not uniform: e.g. transferrin is a predictive factor in two studies, but not in the third. In addition, the number of trials is low and the number of participants in the Gisbert study is rather low. Therefore, no hard conclusions can be drawn at this moment.

Table 14: Overview of trials exploring factors predicting response to IV iron supplementation

Study	Design	Patients	Treatment	Mean total iron dose	N	Response	Factors predictive of response	Factors not predictive of response
Gasché 2001 [42]	open-label multi-centre (4 w)	IBD + IDA Hb≤10.5 g/dl	IV iron sucrose	1200 mg	103	65%*	□ High serum erythropoietin<br>□ High sTfR<br>□ High transferrin	□ Mean corpuscular Hb<br>□ Ferritin<br>□ CRP<br>□ IL-6<br>□ Disease activity
Gisbert 2009 [35]	open-label multi-centre (6 mo)	IBD + IDA Hb<10 g/dl	IV iron sucrose	NR (2x200 mg/week)	22	77%**	/	□ Iron<br>□ Ferritin<br>□ Transferrin saturation<br>□ Transferrin<br>□ Erythrocyte sedimentation rate<br>□ CRP<br>□ Orosomucoid
Iqbal 2011 [43] FERGIcor subanalysis	open-label, multi-centre RCT (12 w)	IBD + IDA	IV ferric carboxymaltose<br><br>IV iron sucrose	max. 3x 500-1000 mg<br><br>max. 11x 200 mg	399	66.1%*<br><br>54.1%*	□ TfS<20%<br>□ Ferritin <30ng/ml<br>□ Transferrin ≥3g/l	□ Use of anti-TNF treatment<br>□ High CRP<br>□ Remission status

*response: Hb increase ≥2.0 g/dl

**response: complete Hb normalisation (male: ≥13 g/dl; female: ≥12 g/dl)

CRP: C-reactive protein; Hb: haemoglobin; IBD: inflammatory bowel disease; IDA: iron deficiency anaemia; IL: interleukin; IV: intravenous; mo: months; NR: not reported; RCT: randomised controlled trial; sTfR: serum transferrin receptor; Tfs: transferrin saturation; TNF: tumour necrosis factor; w: weeks

back to Index

3.3. Guidelines and recommendations on treatment of ID

One of the most important guidelines for treatment of IDA in patients with IBD are those formulated by the international working group [2].
They state that all IBD patients should be assessed for presence of anaemia. Iron supplementation should be initiated when IDA is present (grade A recommendation). The preferred route of iron supplementation in IBD patients is IV, even though many patients respond to oral iron (grade A recommendation). Although IV iron is less convenient, it is more effective, better tolerated and can improve quality of life to a greater extent. Absolute indications for IV iron supplementation are presented in Table 15. Oral iron supplements can be used if the absolute indications for IV iron therapy are not met.

According to the same guidelines, the use of ESA is effective for the treatment of ACD and should be considered if haemoglobin <10.0g/dl or if there is no response to IV iron therapy within 4 weeks (grade B recommendation). ESA treatment should be combined with IV iron therapy (grade A recommendation).

Indication	Definition
Severe anaemia	Haemoglobin <10 g/dl
Intolerance to oral iron	
Inappropriate response to oral iron	[Hb] ↑ ≥ 2 g/dl or reaches normal within 4 weeks
Severe intestinal disease activity	
Concomitant therapy with ESA	
Patient preference	

Table 15: Absolute indications for IV iron supplementation according to [2]
[Hb]: haemoglobin concentration; ESA: erythropoiesis-stimulating agent

The British Society of Gastroenterology (BSG) published an update of their guidelines for IBD in 2011, including recommendations to treat anaemia as it is a common complication of IBD [44]. They indicate that long-term prevention of anaemia by treatment of underlying IBD is primary, but iron replacement is also needed. Supplementation therapy may be with oral iron; however it may not be tolerated well and may exacerbate IBD symptoms. Therefore, IV iron therapy is preferred in patients with poor tolerance to oral iron. In patients with severe anaemia that is not responsive to iron supplementation, treatment with ESA will produce a good response (70-100%); however cost is a limiting factor.

back to Index

The European Crohn's and Colitis Organisation (ECCO) guidelines on ulcerative colitis mention that anaemia and ulcerative colitis deserves greater proactive management by gastroenterologists than it generally receives, because it is associated with substantial impairment of quality of life and refer to the guidelines of the international working group [45,46].
The ECCO guidelines on Crohn's disease also indicate that IDA should be identified and treated [47].

Furthermore, some publications on treating IDA, not specifically for IBD patients, also provide recommendations.
In a general review on IDA, Clark presents the following indications for IV iron supplementation [15]:
- high iron requirements secondary to chronic uncorrectable bleeding or chronic haemodialysis;
- iron malabsorption from gastric resection, atrophic gastritis, or coeliac disease;
- intolerance to oral therapy because of gastrointestinal side effects or poor adherence.

It is emphasised in this paper that for proper management of ID the diagnosis should be accurately validated and the underlying aetiology should be identified.

The recommendations of the BSG for the management of IDA, intended for gastroenterologists and gastrointestinal surgeons are summarised in Table 16 [48].

Recommendation	Grade
All patients with evidence of IDA should have iron supplementation both to correct anaemia and replenish body stores	B
Parenteral iron can be used when oral preparations are not tolerated	C
Blood transfusions should be reserved for patients with or at risk of cardiovascular instability due to the degree of their anaemia	C

Table 16: Recommendations for the management of IDA according to [48]
IDA: iron deficiency anaemia

Some recent algorithms for treatment of IDA in patients with IBD are presented in Figures 11, 12 and 13.

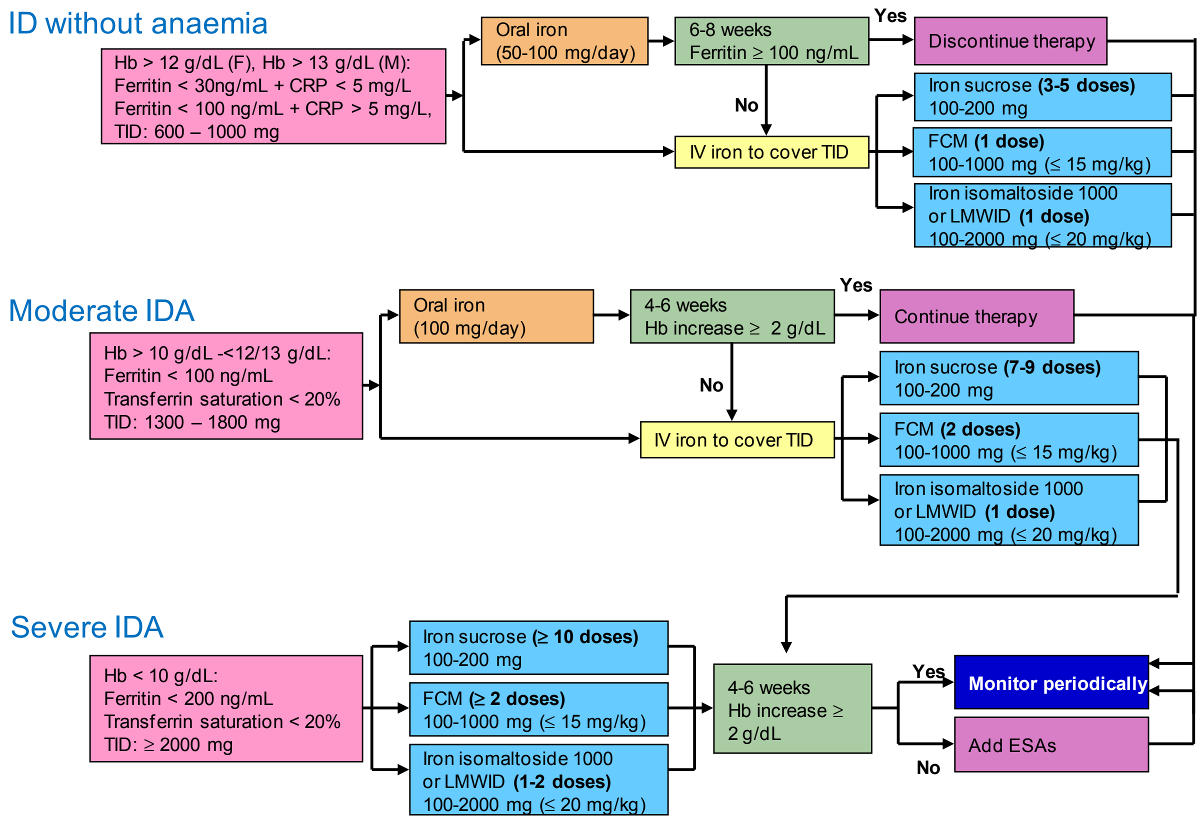

Figure 11: Algorithm for iron supplementation in patients with IBD as presented at UEGW 2010 (Barcelona, 23-27 October 2010); adapted from [22]
CRP: C-reactive protein; ESA: erythropoiesis-stimulating agent; FCM: ferric carboxymaltose; Hb: haemoglobin; ID: iron deficiency; IV: intravenous; LMWID: low molecular weight iron dextran; TID: total iron dose

back to Index

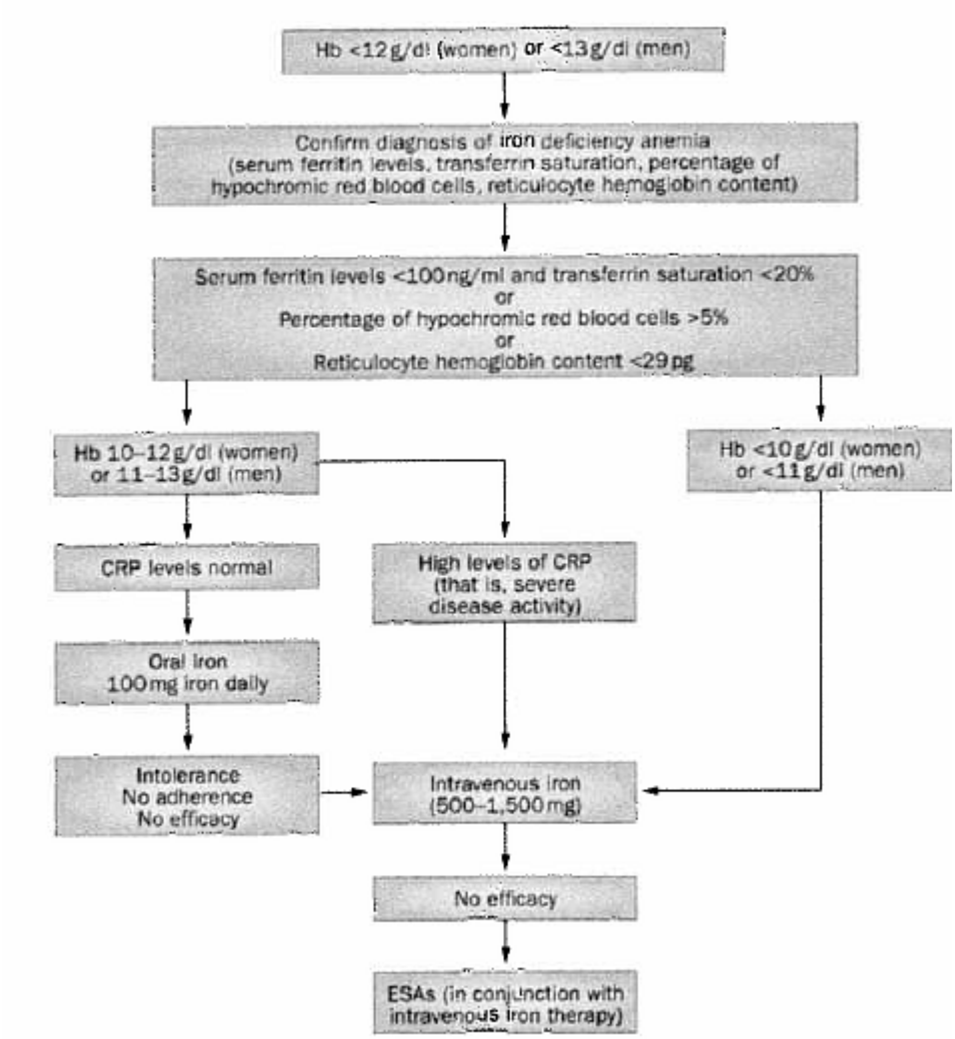

Figure 12: Algorithm for the management of IDA in IBD [16]
CRP: C-reactive protein; ESA: erythropoiesis-stimulating agent; Hb: haemoglobin

back to Index

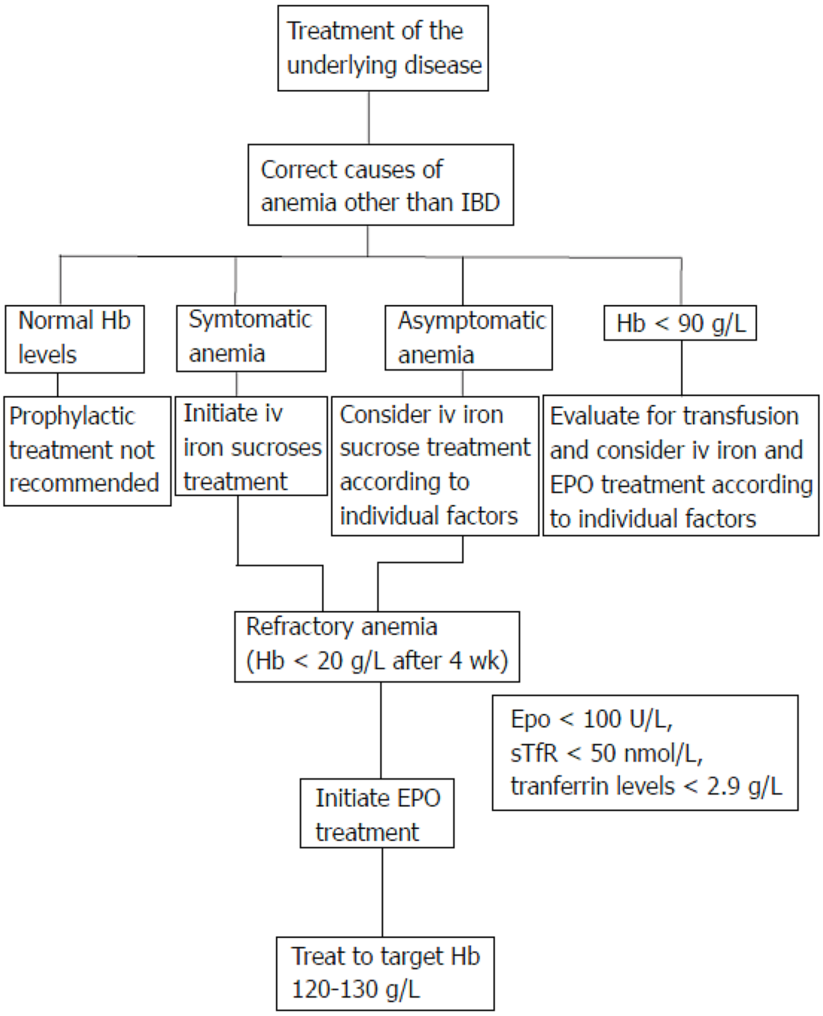

Figure 13: Treatment algorithm for stimulating erythropoiesis in IBD-associated anaemia [20]

EPO: erythropoietin; Hb: haemoglobin; IBD: inflammatory bowel disease; iv: intravenous; sTfR: serum transferrin receptor

4. REFERENCES

[1] Oustamanolakis P, Koutroubakis IE, Kouroumalis EA. Diagnosing anemia in inflammatory bowel disease: beyond the established markers. J Crohns Colitis 2011;5:381-91.

[2] Gasche C, Berstad A, Befrits R et al. Guidelines on the diagnosis and management of iron deficiency and anemia in inflammatory bowel diseases. Inflamm Bowel Dis 2007;13:1545-53.

[3] Gisbert JP, Gomollón F. Common misconceptions in the diagnosis and management of anemia in inflammatory bowel disease. Am J Gastroenterol 2008;103:1299-307.

[4] Goodhand JR, Kamperidis N, Rao A et al. Prevalence and management of anemia in children, adolescents, and adults with inflammatory bowel disease. Inflamm Bowel Dis 2011;epub:doi:10.1002/ibd.21740.

[5] Bager P, Befrits R, Wikman O et al. The prevalence of anemia and iron deficiency in IBD outpatients in Scandinavia. Scand J Gastroenterol 2011;46:304-9.

[6] Wilson A, Reyes E, Ofman J. Prevalence and outcomes of anemia in inflammatory bowel disease: a systematic review of the literature. Am J Med 2004;116(Suppl 7A):44S-9S.

[7] Bergamaschi G, Di Sabatino A, Albertini R et al. Prevalence and pathogenesis of anemia in inflammatory bowel disease. Influence of anti-tumor necrosis factor-□ treatment. Haematologica 2010;95:199-205.

[8] Oustamanolakis P, Koutroubakis IE, Messaritakis I et al. Measurement of reticulocyte and red blood cell indices in the evaluation of anemia in inflammatory bowel disease. J Crohns Colitis 2011;5:295-300.

[9] Gomollón F, Gisbert JP. Anemia and inflammatory bowel diseases. World J Gastroenterol 2009;15:4659-65.

[10] Weiss G, Gasche C. Pathogenesis and treatment of anemia in inflammatory bowel disease. Haematologica 2010;95:175-8.

[11] Gasche C, Lomer MCE, Cavill I, Weiss G. Iron, anaemia, and inflammatory bowel diseases. Gut 2004;53:1190-7.

[12] Theurl I, Aigner E, Theurl M et al. Regulation of iron homeostasis in anemia of chronic disease and iron deficiency anemia: diagnostic and therapeutic implications. Blood 2009;113:5277-86.

[13] Weiss G, Goodnough LT. Anemia of chronic disease. N Engl J Med 2005;352:1011-23.

[14] Oldenburg B, Koningsberger JC, Van Berge Henegouwen GP, Van Asbeck BS, Marx JJM. Iron and inflammatory bowel disease. Aliment Pharmacol Ther 2001;15:429-38.

[15] Clark SF. Iron deficiency anemia. Nutr Clin Pract 2008;23:128-41.

[16] Stein J, Hartmann F, Dignass AU. Diagnosis and management of iron deficiency anemia in patients with IBD. Nat Rev Gastroenterol Hepatol 2010;7:599-610.

back to Index

[17] Oustamanolakis P, Koutroubakis IE. Soluble transferrin receptor-ferritin index is the most efficient marker for the diagnosis of iron deficiency anemia in patients with IBD. Inflamm Bowel Dis 2011;17:E158-E159.

[18] Bermejo F, García-López S. A guide to diagnosis of iron deficiency and iron deficiency anemia in digestive diseases. World J Gastroenterol 2009;15:4638-43.

[19] Ganzoni AM. [Intravenous iron-dextran: therapeutic and experimental possibilities]. Schweiz Med Wochenschr 1970;100:301-3.

[20] Tsiolakidou G, Koutroubakis IE. Stimulating erythropoiesis in inflammatory bowel disease associated anemia. World J Gastroenterol 2007;13:4798-806.

[21] Zhu A, Kaneshiro M, Kaunitz JD. Evaluation and treatment of iron deficiency anemia: a gastroenterological perspective. Dig Dis Sci 2010;55:548-59.

[22] Muñoz M, Gómez-Ramírez S, García-Erce JA. Intravenous iron in inflammatory bowel disease. World J Gastroenterol 2009;15:4666-74.

[23] Chertow GM, Mason PD, Vaage-Nilsen O, Ahlmén J. Update on adverse drug events associated with parenteral iron. Nephrol Dial Transplant 2006;21:378-82.

[24] Bayraktar UD, Bayraktar S. Treatment of iron deficiency anemia associated with gastrointestinal tract diseases. World J Gastroenterol 2010;16:2720-5.

[25] Bhandari S. A hospital-based cost minimization study of the potential financial impact on the UK health care system of introduction of iron isomaltoside 1000. Ther Clin Risk Manag 2011;7:103-13.

[26] de Cid R, Riveira-Munoz E, Zeeuwen PLJ et al. Deletion of the late cornified envelope LCE3B and LCE3C genes as a susceptibility factor for psoriasis. Nat Genet 2009;41:211-5.

[27] Moreno López R, Sicilia Aladrén B, Gomollón García F. Use of agents stimulating erythropoiesis in digestive diseases. World J Gastroenterol 2009;15:4675-85.

[28] Circular of AABB, the American Red Cross, America's Blood Centers, and the Armed Services Blood Program. Available at: http://www.aabb.org/resources/bct/Documents/coi0809r.pdf

[29] Erichsen K, Ulvik RJ, Grimstad T et al. Effects of ferrous sulphate and non-ionic iron-polymaltose complex on markers of oxidative tissue damage in patients with inflammatory bowel disease. Aliment Pharmacol Ther 2005;22:831-8.

[30] Erichsen K, Ulvik RJ, Nysaeter G et al. Oral ferrous fumarate or intravenous iron sucrose for patients with inflammatory bowel disease. Scand J Gastroenterol 2005;40:1058-65.

[31] Schröder O, Mickisch O, Seidler U et al. Intravenous iron sucrose versus oral iron supplementation for the treatment of iron deficiency anemia in patients with inflammatory bowel disease--a randomized, controlled, open-label, multicenter study. Am J Gastroenterol 2005;100:2503-9.

[32] Kulnigg S, Stoinov S, Simanenkov V et al. A novel intravenous iron formulation for treatment of anemia in inflammatory bowel disease: the ferric carboxymaltose (FERINJECT®) randomized controlled trial. Am J Gastroenterol 2008;103:1182-92.

[33] Lindgren S, Wikman O, Befrits R et al. Intravenous iron sucrose is superior to oral iron sulphate for correcting anaemia and restoring iron stores in IBD patients: a randomized, controlled, evaluator-blind, multicentre study. Scand J Gastroenterol 2009;44:838-45.

[34] Wells CW, Lewis S, Barton JR, Corbett S. Effects of changes in hemoglobin level on quality of life and cognitive function in inflammatory bowel disease patients. Inflamm Bowel Dis 2006;12:123-30.

[35] Gisbert JP, Bermejo F, Pajares R et al. Oral and intravenous iron treatment in inflammatory bowel disease: hematological response and quality of life improvement. Inflamm Bowel Dis 2009;15:1485-91.

[36] Khalil A, Goodhand JR, Wahed M et al. Efficacy and tolerability of intravenous iron dextran and oral iron in inflammatory bowel disease: a case-matched study in clinical practice. Eur J Gastroenterol Hepatol 2011;23:1029-35.

[37] Evstatiev R, Marteau P, Iqbal T et al. FERGIcor, a randomized controlled trial on ferric carboxymaltose for iron deficiency anemia in inflammatory bowel disease. Gastroenterology 2011;141:846-53.e2.

[38] Schreiber S, Howaldt S, Schnoor M et al. Recombinant erythropoietin for the treatment of anemia in inflammatory bowel disease. N Engl J Med 1996;334:619-23.

[39] Gasché C, Dejaco C, Waldhoer T et al. Intravenous iron and erythropoietin for anemia associated with Crohn disease. A randomized, controlled trial. Ann Intern Med 1997;126:782-7.

[40] Gasché C, Reinisch W, Lochs H et al. Anemia in Crohn's disease. Importance of inadequate erythropoietin production and iron deficiency. Dig Dis Sci 1994;39:1930-4.

[41] Gasche C, Dejaco C, Reinisch W et al. Sequential treatment of anemia in ulcerative colitis with intravenous iron and erythropoietin. Digestion 1999;60:262-7.

[42] Gasche C, Waldhoer T, Feichtenschlager T et al. Prediction of response to iron sucrose in inflammatory bowel disease-associated anemia. Am J Gastroenterol 2001;96:2382-7.

[43] Iqbal T, Evstatiev R, Chopey K, Harjunpää J, Gasche C. Significant benefit of ferric carboxymaltose in IBD-associated iron deficiency anaemia is independent of patient baseline characteristics. J Crohns Colitis 2011;5:S96 (abs. P200)-S97 (abs. P200).

[44] Mowat C, Cole A, Windsor A et al. Guidelines for the management of inflammatory bowel disease in adults. Gut 2011;60:571-607.

[45] Biancone L, Michetti P, Travis S et al. European evidence-based Consensus on the management of ulcerative colitis: special situations. J Crohns Colitis 2008;2:63-92.

[46] Travis SPL, Stange EF, M. Lémann M et al. European evidence-based Consensus on the management of ulcerative colitis: Current management J Crohns Colitis 2008;2:24-62.

[47] Dignass A, van Assche G, Lindsay JO et al. The second European evidence-based consensus on the diagnosis and management of Crohn's disease: current management. J Crohns Colitis 2010;4:28-62.

[48] Goddard AF, James MW, McIntyre AS, Scott BB, on behalf of the British Society of Gastroenterology. Guidelines for the management of iron deficiency anaemia. Gut 2011;60:1309-16.
